# Viral protease binds to nucleosomal DNA and cleaves nuclear cGAS that attenuates type I interferon

**DOI:** 10.1128/mbio.03395-24

**Published:** 2025-02-25

**Authors:** Lei Wu, Ya Yan, Ye Yuan, Zhenchao Zhao, Weiyu Qu, Xiangyu Huang, Haiwei Wang, Pingwei Li, Xin Li

**Affiliations:** 1 National Key Laboratory of Veterinary Public Health and Safety, College of Veterinary Medicine, China Agricultural University, Beijing, China; 2 Key Laboratory of Animal Epidemiology of the Ministry of Agriculture and Rural Affairs, College of Veterinary Medicine, China Agricultural University, Beijing, China; 3 State Key Laboratory of Animal Disease Control, Harbin Veterinary Research Institute, Chinese Academy of Agricultural Sciences, Harbin, China; 4 Department of Biochemistry and Biophysics, Texas A&M University, College Station, Texas, USA; The Catholic University of America, Washington, DC, USA; The Catholic University of America, Washington, DC, USA

**Keywords:** nuclear cGAS, Seneca Valley virus, 3C protease, nucleosomal DNA

## Abstract

Nuclear cyclic GMP-AMP synthetase (cGAS) binds to nucleosome with high affinity to prevent its activation by self-DNA. Upon stimulation with double-stranded DNA, cGAS is activated and translocates from the nucleus to the cytoplasm, guided by its N-terminal domain. However, it remains unclear whether viruses can hijack cGAS translocation and regulate its activation. Here, we discovered that the protease 3C of picornavirus Seneca Valley virus (SVV) translocates from the cytoplasm to the nucleus upon viral infection and binds to nuclear DNA. Protease 3C specifically cleaves histone H2A while leaving other histone proteins unaffected. Additionally, DNA binding enhances the protease 3C’s ability to cleave nuclear cGAS, leading to its retention in the nucleus. This, in turn, suppresses the induction of type I interferon (IFN-I) following poly(dA:dT) stimulation. These findings reveal a novel mechanism by which a viral protease binds nuclear DNA, cleaves nuclear cGAS and histone H2A, and thereby mislocalizes cGAS, facilitating immune evasion.

**IMPORTANCE:**

Cyclic GMP-AMP synthetase (cGAS) is robustly expressed in the nucleus and tightly tethered by chromatin to prevent its activation with self-DNA. During stimulation or infection, nuclear cGAS is activated and translocates from the nucleus to the cytoplasm. However, the viral strategies specifically targeting nuclear cGAS are completely unexplored. Here, we discovered that protease 3C of Seneca Valley virus translocates from the cytoplasm to the nucleus upon viral infection, binds to nuclear DNA, and specifically cleaves H2A. Furthermore, DNA binding to 3C enhances the cleavage of nuclear cGAS within its N-terminal domain. The hindrance of cGAS translocation from the nucleus to the cytoplasm results in the suppression of IFN-I induction and leads to immune evasion. This work uncovers a unique mechanism wherein a viral protease binds to nuclear DNA and cleaves nuclear cGAS and histone H2A, leading to viral evasion of cGAS-mediated immune restriction.

## INTRODUCTION

Cyclic GMP-AMP synthetase (cGAS) senses misplaced genomic, mitochondrial, and microbial double-stranded DNA (dsDNA), resulting in its activation and the synthesis of the cyclic dinucleotide 2′3′-cGAMP (1). Serving as a second messenger, cGAMP mediates the activation of innate immune responses through the stimulator of interferon genes (STING) and downstream signaling to induce the expression of type I interferons (IFN-I) (2, 3). cGAS consists of a positively charged disordered N-terminal domain and a C-terminal catalytic domain (4), which play a key role in binding DNA and catalyzing the synthesis of cGAMP (5, 6). In recent years, there has been growing research interest in the function of N-terminal domain of cGAS. Due to the disordered nature of the cGAS N-terminal domain across species, several studies have demonstrated its critical role in promoting liquid-phase condensation with DNA, which is vital for activating cGAS (7). Furthermore, the N-terminal domain of cGAS interacts with phosphatidylinositol 4,5-bisphosphate (PI(4,5)P2), a lipid found in the plasma membrane, facilitating its localization to the membrane (8). Meanwhile, cGAS N-terminal domain is hyperphosphorylated by mitotic kinases to prevent the activation of cGAS by the chromatin during mitosis (9).

Accumulating evidence indicates that cGAS is localized in both cytoplasm and nucleus, with a predominant distribution in the nucleus (10–13). Recent studies have shown that the structural basis for this inactivation involves nuclear cGAS being tightly bound to the acidic patch of histone H2A and H2B, which blocks the DNA-binding site B of cGAS (14–16). In the event of DNA damage, nuclear cGAS is recruited to double-strand breaks, interacts with PARP1, and suppresses DNA homologous recombination repair, thereby promoting tumorigenesis (17). Moreover, nuclear cGAS plays a crucial role in stabilizing replication forks to maintain genome integrity (18). Recent studies have shown that nuclear cGAS can catalyze the synthesis of cGAMP and trigger the innate immune activation of dendritic cells (DCs), although cGAMP levels are 200-fold lower than those following transfection with exogenous DNA (12). In the nucleus, cGAS was also found to be activated by human immunodeficiency virus (HIV) through interactions with NONO (non-POU domain-containing octamer-binding protein) in DCs and macrophages (19). Additionally, nuclear cGAS recruits protein arginine methyltransferase 5 (Prmt5), facilitating its binding to the promoters and enhancers, thereby enhancing type I IFN production (20). Reports also indicate that the cytoplasmic cGAS is exported from the nucleus upon stimulation (21), and nuclear soluble cGAS is activated by dsDNA during herpes simplex virus I (HSV-1) infection, which leads to production of cGAMP and IFN-I (22). Viruses have developed multiple strategies to evade or counteract cGAS recognition, including autophagy-dependent degradation (23, 24), stabilization of caspase-1 to cleave cGAS (25), competitive binding to dsDNA (26, 27), and direct cleavage of cGAS (28). However, there are no reports of nuclear cGAS being cleaved by a virus-encoded protease.

The Seneca Valley virus (SVV) is a non-enveloped, single-stranded, positive-sense RNA virus belonging to the *Picornaviridae* family. It has been shown that SVV has oncolytic properties and shows significant potential as an oncolytic virus (29). SVV 3C protease is critical for viral polypeptide processing and evasion of host immunity. In our previous study, we found that SVV 3C specifically cleaves porcine cGAS (pcGAS), which suppresses mitochondrial DNA-mediated innate immune sensing (28). In addition to SVV, *picornaviridae* also includes poliovirus (PV), human rhinovirus (HRV), encephalomyocarditis virus (EMCV), enterovirus 71 (EV-A71), and coxsackievirus. To date, PV encoded protease-polymerase precursor 3CD has been reported to enter the nucleus and induce transcriptional shutdown in host cells (30). HRV protease 3C and its precursor form, known as 3CD, have also been detected in the nucleus where they modulate nucleocytoplasmic transport processes (31, 32). EV-A71 3C has also been observed in the nucleus during viral infection (33). However, it is not clear whether SVV 3C enters the nucleus and regulates similar cellular processes.

In this study, we observed nuclear DNA leakage into the cytosol following SVV infection, specifically caused by SVV protease 3C. We demonstrated that multiple picornaviruses 3C could enter the nucleus and identified the N-terminal 1-20 amino acids as key residues for the nuclear translocation of SVV 3C. Mechanistically, SVV 3C cleaves histone H2A, disrupting nucleosome function, and binds to nucleosomal DNA. In addition, nuclear cGAS was cleaved at the N-terminus following 3C transfection or SVV infection. Interestingly, this cleavage was enhanced by the addition of interferon stimulatory DNA (ISD) or nucleosomal DNA in a cell-free system, which inhibited the translocation of pcGAS from the nucleus to the cytosol upon poly(dA:dT) stimulation, thereby suppressing cGAS activity. In summary, our findings reveal that SVV protease 3C translocates to the nucleus, where it binds to nucleosomal DNA, cleaves histone H2A and nuclear cGAS to reduce the production of IFN-I, highlighting a novel viral strategy to hijack nuclear cGAS activation.

## RESULTS

### SVV infection induces the translocation of protease 3C into the nucleus and leads to nuclear DNA leakage

**Fig 1:**
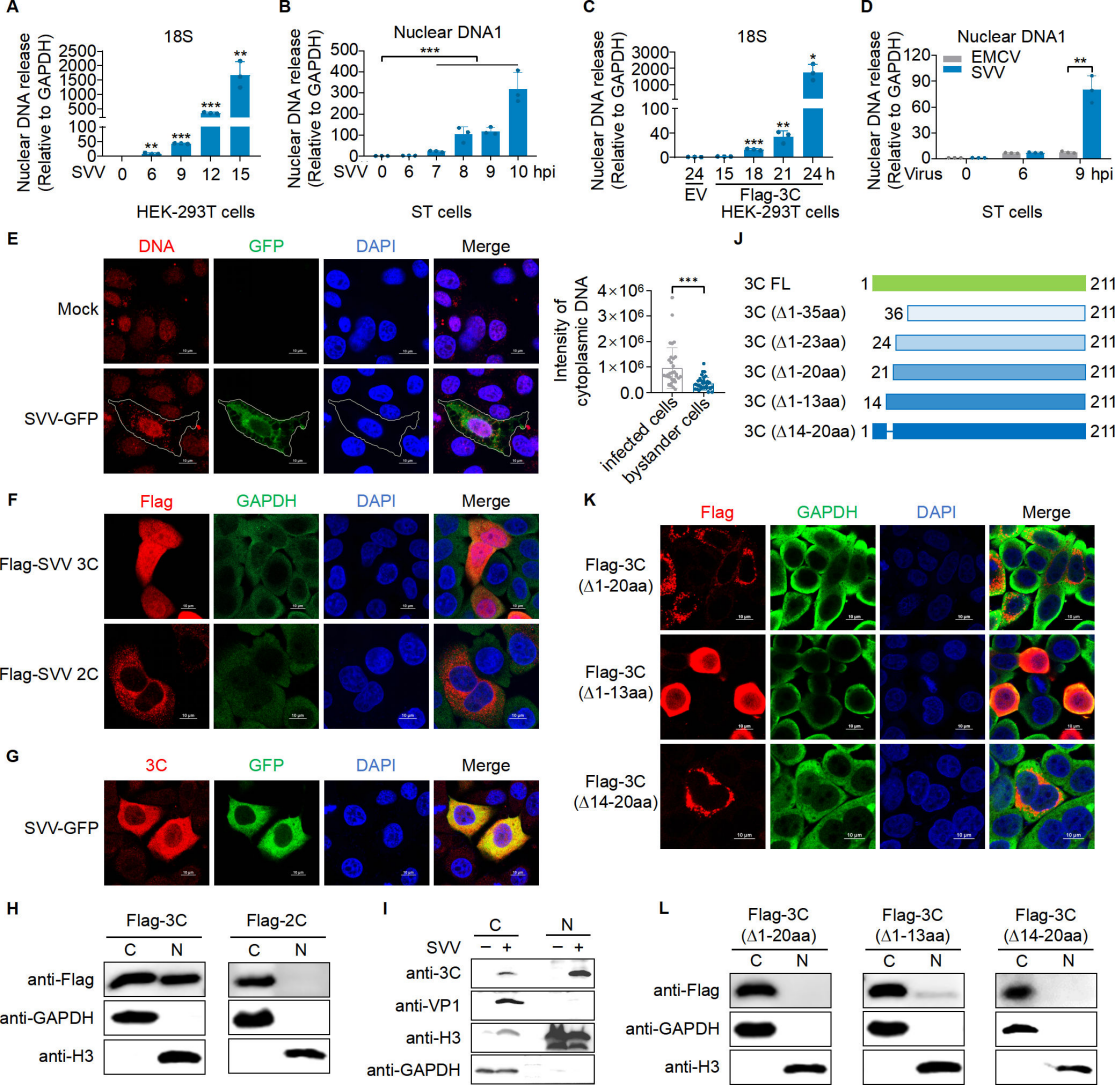
SVV infection induces protease 3C translocation into the nucleus and causes nuclear DNA leakage. (**A**) qPCR analysis of 18S in the cytoplasm of HEK-293T cells infected with SVV at an MOI of 10 for 0, 6, 9, 12, 15, or 18 h. (**B**) qPCR analysis of nuclear DNA in the cytoplasm of ST cells infected with SVV at an MOI of 10 for 0, 6, 7, 8, 9, and 10 h. (**C**) qPCR analysis of 18S in the cytoplasm of HEK-293T cells mock transfected or transfected with a plasmid expressing Flag-3C for 15, 18, 21, or 24 h. (**D**) qPCR analysis of nuclear DNA in the cytoplasm of ST cells (MOI 10). (**E**) Confocal microscopy analysis of DNA in mock ST cells or ST cells infected with SVV-GFP at an MOI of 10 for 9 h. The graph in the right panel shows the quantification of cytosolic DNA. (**F**) Localization analysis of 3C or 2C in HeLa cells transfected with a plasmid expressing 3C or 2C for 18 h using confocal microscopy. (**G**) Confocal microscopy analysis of 3C localization in ST cells infected with SVV-GFP (MOI 10) for 9 h. (**H**) Western blot analysis of 3C or 2C in HeLa nuclear and cytoplasmic fractions corresponding to (**F**). The nuclear and cytoplasmic fractions were separated using Nuclear and Cytoplasmic Extraction Reagents. The purity of the respective fractions was assessed by detecting nuclear histone H3 and cytoplasmic GAPDH. Scale bars, 10 µm. (I) Western blot analysis of 3C in nuclear and cytoplasmic fractions of ST cells infected with SVV (MOI 10) for 12 h. (**J**) Schematic diagram of plasmid 3C truncation or mutation. (**K**) Confocal microscopy analysis of 3C localization in HeLa cells transfected with plasmids encoding different truncated forms of 3C. Scale bars, 10 µm. (**L**) Western blot analysis of 3C in the nucleus and cytoplasm of HeLa cells transfected with plasmids encoding Flag-3C with amino acids 1-20, 1-13, or 14-20 deletions corresponding to (**K**). Scale bars, 10 µm (**E–G, K**). Results are representative of three biological replicates. Means ± SD are shown in (**A–D**) (*n* = 3) and E (*n* = 33). Two-tailed unpaired *t*-test was used for the statistical analysis, *P < 0.1, **P < 0.01, ***P < 0.001.

An increasing number of studies have shown that RNA viruses, such as dengue virus (DENV), influenza A virus (IAV), EMCV, Zika virus (ZIKV), cause the leakage of mitochondrial DNA (mtDNA) in the cytoplasm upon infection (25, 34). Our previous work has shown that SVV infection also leads to mtDNA leakage, which in turn activates cGAS (28). Interestingly, norovirus infection was recently reported to induce the leakage of both mtDNA and nuclear DNA into the cytoplasm (35). Building on these findings, we investigated whether SVV infection also causes the leakage of nuclear DNA into the cytoplasm. To explore this, we infected various cell types, including human embryonic kidney (HEK-293T), porcine kidney-15 (PK-15), and swine testis (ST) cells with SVV (Fig. 1A and B; Fig. S1A and B). After infection, we separated the cytoplasm and nucleus using an isolation kit, and quantified leaked nuclear DNA in the cytoplasm using RT-qPCR with specific primers for 18S and Myc. Our data showed that the levels of 18S and Myc were significantly elevated 9 h post-infection in HEK-293T cells (Fig. 1A; Fig. S1A). Similarly, nuclear DNA levels were obviously increased in the cytoplasm of ST and PK-15 cells (Fig. 1B; Fig. S1B), suggesting widespread nuclear DNA leakage in both human and porcine cells upon SVV infection. It has been reported that SVV 2C and 3C induce apoptosis and cleave poly (ADP ribose) polymerase 1 (PARP1) (36), which plays a key role in DNA repair and maintaining genomic stability (37). Given that the protease of PV and HRV can translocate to the nucleus, we speculated whether SVV proteases 2C or 3C also possess this capability. Interestingly, we found that the levels of 18S and Myc significantly increased in the cytoplasm following 3C transfection, but this increase was not observed with 2C transfection (Fig. S1C and D). Subsequently, the levels of 18S and Myc in the cytoplasm were significantly elevated 18 h post-transfection in HEK-293T cells (Fig. 1C; Fig. S1E). Notably, the catalytically inactive mutant H48A/C160A 3C significantly reduced nuclear DNA leakage compared to wild-type 3C (Fig. S1F). To determine whether EMCV infection also induces nuclear DNA leakage, we infected ST cells with SVV and EMCV. The results showed that SVV induced significantly higher nuclear DNA leakage than EMCV 9 h post-infection (Fig. 1D). To rule out the lower infectivity as a cause for the difference, we assessed the infectivity of both SVV and EMCV through dsRNA staining. The results indicated that the infectivity of EMCV was similar to or even higher than that of SVV (Fig. S1G). To further confirm the leakage of nuclear DNA upon SVV infection, we infected ST cells with GFP-SVV and stained them with anti-DNA antibody. Fluorescence microscopy revealed a higher level of DNA (red) in the cytoplasm of infected cells compared to mock cells (Fig. 1E). The extensive leakage of nuclear DNA into the cytosol suggests significant instability and loss of function in the nucleus.

To investigate whether protease 3C can translocate to and damage the nucleus, we assessed the subcellular distribution of SVV 3C using confocal microscopy. After transfection in HeLa cells, 3C was significantly present in both the cytoplasm and nucleus, whereas 2C, serving as a negative control, was only detected in the cytoplasm (Fig. 1F). Additionally, in ST cells infected with SVV-GFP, the fluorescence of SVV 3C protein was observed in both the cytoplasm and nucleus (Fig. 1G). These results were further confirmed at the protein level by Western blot analysis (Fig. 1H and I). To investigate the process of 3C entering the nucleus, we infected ST and HEK-293T cells with SVV and analyzed the distribution of 3C in the cytoplasm and nucleus at different time points post-infection. We found that 3C was already present in the cytoplasm and nucleus as soon as it became detectable, and the amount of 3C in the nucleus gradually increased as the infection progressed (Fig. S2A and B). We also observed some non-specific bands in the cytoplasm, which may explain the brighter red fluorescence in the cytoplasm in Figure 1G. Protease 3C from PV, EV-A71, and HRV have been reported to localize in the nucleus (30–33). Interestingly, we found that 3C from EV-A71, EMCV, CVB3, PV, and HRV also translocated to the nucleus after transfection (Fig. S2C through F). Notably, SVV 3C lacks a nuclear localization signal (NLS), prompting us to screen for key residues essential for its nuclear translocation. Considering that 3C contains 211 residues with crucial catalytic residues at H48, D84, and C160, we constructed N-terminal truncations to avoid affecting enzymatic activity (Fig. 1J). The deletion of residues 1-35 (3C ∆1-35) or 1-23 (3C ∆1-23) abolished nuclear translocation 18 h post-transfection in HeLa cells (Fig. S3). To further investigate, we transfected HeLa cells with 3C mutants ∆1-20, ∆1-13, and ∆14-20 amino acids (aa), respectively. The results showed that the 3C ∆1-20 and ∆14-20 mutants lost the ability to translocate to the nucleus, while the 3C ∆1-13 mutant exhibited a significant reduction in nuclear translocation, although a small amount still entered the nucleus (Fig. 1K and L). Taken together, these results demonstrated that SVV infection leads to significant nuclear DNA accumulation in the cytoplasm. Unlike 2C, SVV protease 3C translocates to the nucleus, a process facilitated by its N-terminal residues 1-20. Additionally, our findings confirm that other picornaviruses, including EV-A71, EMCV, CVB3, PV, and HRV 3C, also translocate to the nucleus.

### SVV protease 3C specifically cleaves H2A

**Fig 2:**
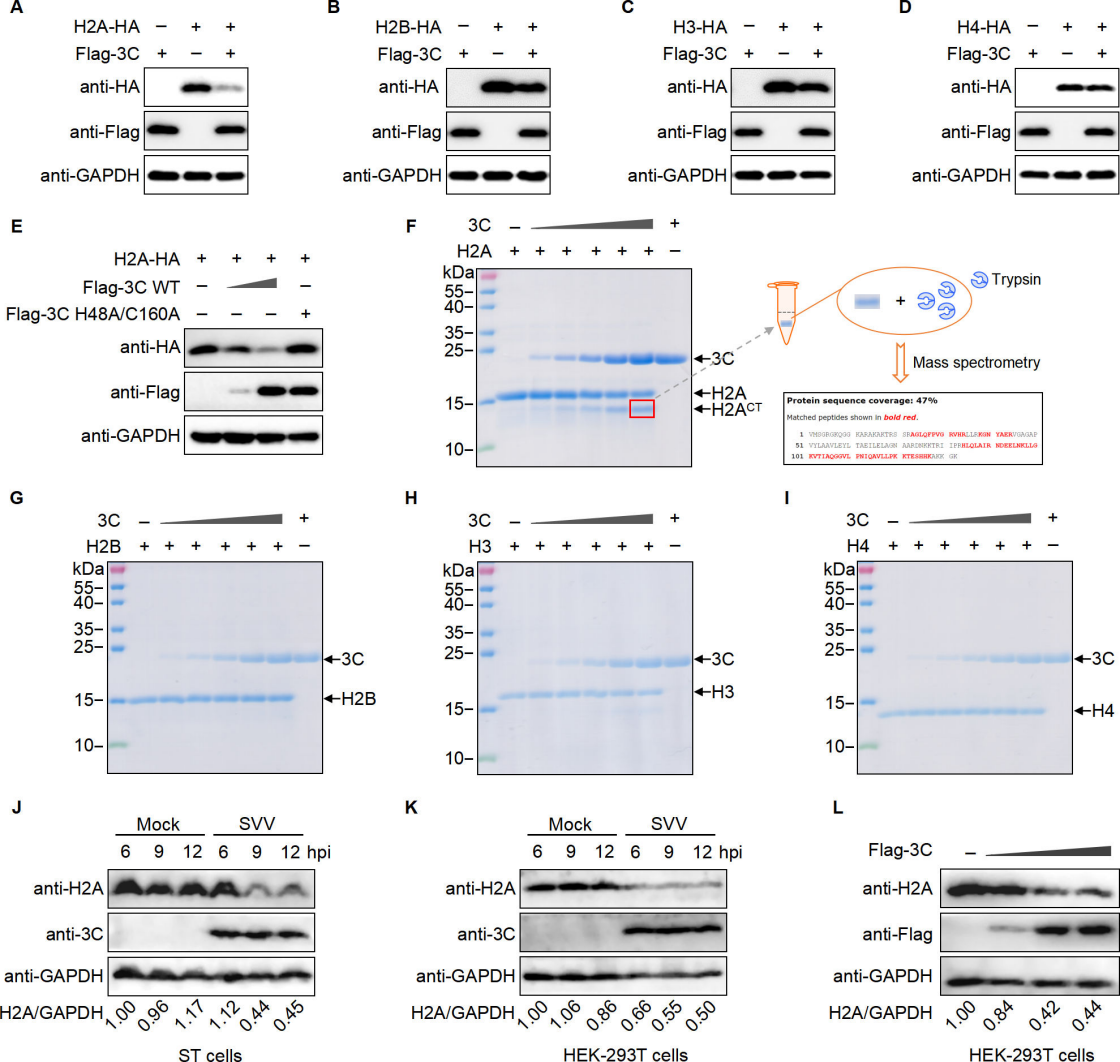
SVV protease 3C specifically cleaves H2A. (**A–D**) Western blot analysis of the expression levels of different histone proteins in HEK-293T cells transfected with a plasmid encoding 3C, together with a plasmid encoding H2A (**A**), H2B (**B**), H3 (**C**), or H4 (**D**) for 24 h. (**E**) Western blot analysis of H2A cleavage by transiently expressing H2A and 3C (wild-type or mutant 3C) in HEK-293T cells. (**F–I**) SDS-PAGE analysis of *in vitro* cleavage of H2A (**F**), H2B (**G**), H3 (**H**), and H4 (**I**) in a 25 µL reaction containing 10 µg of Histone-6×His recombinant protein and varying amounts of purified recombinant 3C protein (1, 5, 10, 15, or 20 µg) incubated for 2 h at 37°C, followed by protein sequence analysis of the cleaved H2A band using mass spectrometry. (**J and K**) Western blot analysis of endogenous H2A in ST (**J**) or HEK-293T (**K**) cells treated with or without SVV (MOI 10) for 6, 9, or 12 h. (**L**) Western blot analysis of endogenous H2A in HEK-293T cells transfected with a plasmid encoding Flag-3C.

Nucleosomes play a crucial role in chromosome stability, gene expression regulation, and DNA replication and repair (38). The nucleosome core proteins include histone H2A, H2B, H3, and H4. Given reports that foot-and-mouth disease virus (FMDV) 3C cleaves H3, and the deleted part of the histone H3 corresponds to the presumed regulatory domain involved in the regulation of transcriptionally active chromatin in eukaryotes (39). Given that SVV 3C translocates to the nucleus, we tested whether SVV 3C could also cleave histone proteins. HEK-293T cells were transfected with a plasmid encoding SVV 3C, together with a plasmid encoding H2A, H2B, H3, or H4 for 24 h. The expression of H2A was significantly reduced after co-transfection with 3C (Fig. 2A), while the expression of H2B, H3, and H4 was not affected by 3C (Fig. 2B through D). To determine whether the decrease of H2A levels depended on the enzymatic activity of 3C, we co-transfected HEK-293T cells with plasmids encoding H2A along with either wild-type (WT) or inactive 3C (H48A/C160A). The results showed a dose-dependent decrease in H2A protein levels with WT 3C but not with inactive 3C (H48A/C160) 24 h post-transfection (Fig. 2E), indicating that the enzymatic activity of 3C is crucial for the cleavage of H2A. To confirm whether H2A was cleaved by 3C *in vitro*, recombinant proteins of H2A, H2B, H3, and H4 were expressed in *Escherichia coli* and then the inclusion bodies were refolded and purified by gel filtration. The reaction system including purified SVV 3C with purified H2A, H2B, H3, or H4 was incubated in the HEPES buffer for 2 h, respectively. The results showed that only H2A, but not H2B, H3, or H4, was specifically cleaved by SVV 3C *in vitro* (Fig. 2F through I), consistent with the transfection data in HEK-293T cells. The cleaved band was excised from the SDS-PAGE gel and analyzed by mass spectrometry, which confirmed the cleavage of H2A by 3C (Fig. 2F). In addition, phylogenetic alignment revealed high conservation of H2A across species (Fig. S4A and B), indicating the possibility that 3C could also cleave human H2A. To further confirm the endogenous cleavage, ST and HEK-293T cells were infected by SVV or transfected by 3C plasmid. The endogenous protein level of H2A was significantly reduced in ST and HEK-293T cells either upon infection or transfection (Fig. 2J through L ). The nuclear proteins are primarily involved in DNA replication, transcription, and repair. To test whether 3C affects nuclear proteins, nucleosomes were isolated under physiological conditions and incubated with a recombinant 3C protein at 37°C for 2 h. The protein bands ranging from 100 to 180 kDa were significantly reduced with increasing doses of 3C (Fig. S5A and B). Mass spectrometry was then performed to identify the degraded proteins, revealing that these proteins are involved in DNA replication, transcription, repair, and the maintenance of nucleosome stability (Fig. S5C). Collectively, SVV protease 3C disrupts nucleosomes and specifically cleaves H2A.

### SVV protease 3C binds to the nucleosomal DNA

**Fig 3:**
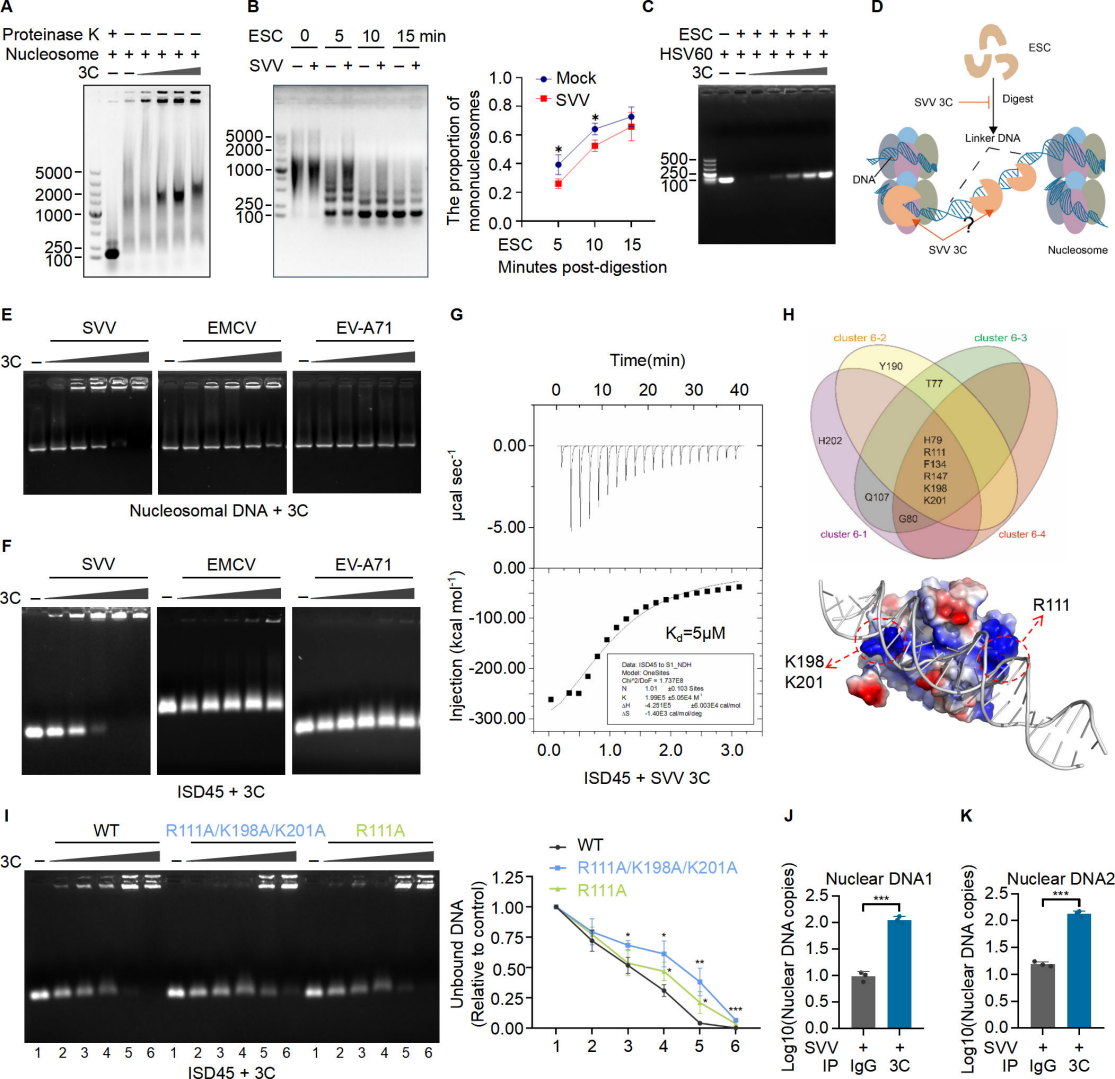
SVV protease 3C binds to the nucleosomal DNA. (**A**) DNA-binding analysis of recombinant protein 3C in a reaction containing different amounts of purified recombinant 3C protein (10, 15, 20, or 25 µg) with the nucleosomes extracted from ST cells for 10 min on ice followed by electrophoretic mobility shift assay (EMSA). (**B**) Detection of the efficiency of Enzymatic Shearing Cocktail (ESC) digestion for nucleosomes. Nucleosomes were extracted from mock ST cells or ST cells infected with SVV (MOI 20) for 9 h and then incubated with ESC for 0, 5, 10, or 15 min at 37°C. The digested nucleosomes were purified, and the nucleosomal DNA was detected using agarose gel electrophoresis. The proportion of mononucleosome produced by ESC digestion was quantified with ImageJ. (**C**) HSV60 and the recombinant protein 3C were mixed at molar ratios of 1:2, 1:4, 1:8, 1:16, and 1:32, followed by incubation with ESC at 37°C for 5 min, after which EDTA was added to stop the reaction. The reaction products were treated with Protease K and then subjected to agarose gel electrophoresis. (**D**) Diagram illustrating how SVV 3C limits ESC digestion of linker DNA. (**E and F**) DNA-binding analysis of recombinant 3C proteins of SVV, EMCV, or EV-A71 with increasing protein concentrations in a reaction containing the nucleosomal DNA extracted from ST cells (**E**) or 45 bp interferon stimulatory DNA (ISD45) (**F**) by EMSA. (**G**) Detection of 3C binding to ISD45 by isothermal titration calorimetry (ITC). (**H**) The binding of SVV 3C to DNA was simulated using HADDOCK2.4 (bottom), and the binding sites of 3C to DNA in cluster 6 with the best score were identified (top). (**I**) DNA-binding analysis of recombinant WT or mutant 3C protein as in the (**F**). (**J and K**) Absolute quantification of nuclear DNA binding to 3C in ST cells infected with SVV (MOI 20) for 9 h by chromatin immunoprecipitation qPCR (ChIP-qPCR). Results are representative of three biological replicates. Means ± SD are shown in (**J and K**) (*n* = 3). Two-tailed unpaired *t*-test was used for the statistical analysis, *P < 0.1, **P < 0.01, ***P < 0.001.

Nucleosome is the smallest subunit of chromatin and contains 147 base pairs of DNA wrapped around an octamer of core histone proteins including H2A, H2B, H3, and H4 (15). To probe the interaction between 3C and nucleosome, we isolated mononucleosome from ST cells. Native gel electrophoresis displayed smeared bands, indicative of large complexes of histone proteins with chromatin DNA. When the histone proteins were digested with proteinase K, nucleosomal DNA migrated to the bottom of the native gel. After the incubation of nucleosomes with 3C protease, nucleosomal DNA migrated more slowly in a dose-dependent manner, suggesting the interaction between 3C and nucleosome (Fig. 3A). To examine the impact of 3C on nucleosome stability, we employed an Enzymatic Shearing Cocktail (ESC) that digests linker DNA between adjacent nucleosomes. ST cells were infected with SVV, after which nucleosomes were isolated from both the SVV-infected and mock-infected groups. The smeared bands appeared darker in the SVV-infected group compared to the mock group at both 5 and 10 min (Fig. 3B), suggesting that 3C likely interacts with linker DNA and protects nucleosomes from digestion by ESC. To further investigate this conclusion, we conducted *in vitro* experiments. Given that linker DNA is approximately 60 bp, we selected a 60 bp herpes simplex virus DNA analog (HSV60) for simulation. HSV60 was incubated with varying doses of 3C recombinant protein or without 3C protein, followed by the addition of ESC. As expected, 3C inhibited the digestion of HSV60 by ESC in a dose-dependent manner (Fig. 3C). As a control, we verified that 3C did not cleave ESC by incubating 3C and ESC recombinant proteins *in vitro* followed by SDS-PAGE analysis (Fig. S6A). To facilitate understanding, we illustrated the action process of 3C in a diagram (Fig. 3D). In addition, we found that 3C inhibited the digestion of HSV60 by ESC at different time points *in vitro*, with the inhibitory effect of 3C on ESC diminishing over the reaction time (Fig. S6B), consistent with the results in Figure 3B. To test whether SVV, EMCV, or EV-A71 3C interacts with nucleosomal DNA, we incubated purified nucleosomal DNA with 3C protein *in vitro* and assessed the interaction using an electrophoretic mobility shift assay (EMSA). Interestingly, the results showed SVV and EMCV 3C interact with nucleosomal DNA, significantly inhibiting nucleosomal DNA migration in a dose-dependent manner. However, SVV 3C demonstrated a significantly stronger binding ability to nucleosomal DNA compared to EMCV 3C (Fig. 3E), while EV-A71 3C did not bind to nucleosomal DNA. Furthermore, we incubated recombinant 3C proteins from SVV, EMCV, or EV-A71 with 45 bp interferon stimulatory DNA (ISD45) and tested the binding interactions by EMSA. Surprisingly, only SVV 3C protein showed a significant dose-dependent binding to ISD45 (Fig. 3F). To confirm the direct interaction between SVV 3C and dsDNA, we performed an isothermal titration calorimetry (ITC) assay, which revealed a binding affinity of approximately 5 µM between 3C protein and ISD45 dsDNA (Fig. 3G). Furthermore, we simulated the binding of SVV 3C with DNA by HADDOCK2.4, with the results from cluster 6 displayed based on the best score (Table S1). Cluster 6 contained four groups of simulated structures. In each group, the DNA-binding sites of 3C were analyzed by a Venn diagram, which identified H79, R111, F134, R147, K198, and K201 as common DNA-binding sites in four groups with these structural models (Fig. 3H). To verify these results, we expressed R111A and R111A/K198A/K201A mutant proteins in *E*. *coli* and purified the recombinant proteins (Fig. S7A and B). DNA-binding studies using EMSA showed that the mutant proteins exhibited reduced interactions with ISD45 compared to WT 3C (Fig. 3I). To provide a physiological context, we infected ST cells with SVV and performed anti-SVV 3C pull-down experiments. Nuclear DNA was associated with SVV 3C but not with anti-IgG control, as determined by amplifying bound DNA with nuclear DNA-specific primers (Fig. 3J and K). These findings demonstrate that SVV protease 3C directly binds to DNA under various conditions, including in cells, cell-free system, and during viral infection.

### SVV 3C co-localizes with and cleaves nuclear cGAS within its N-terminal domain

**Fig 4:**
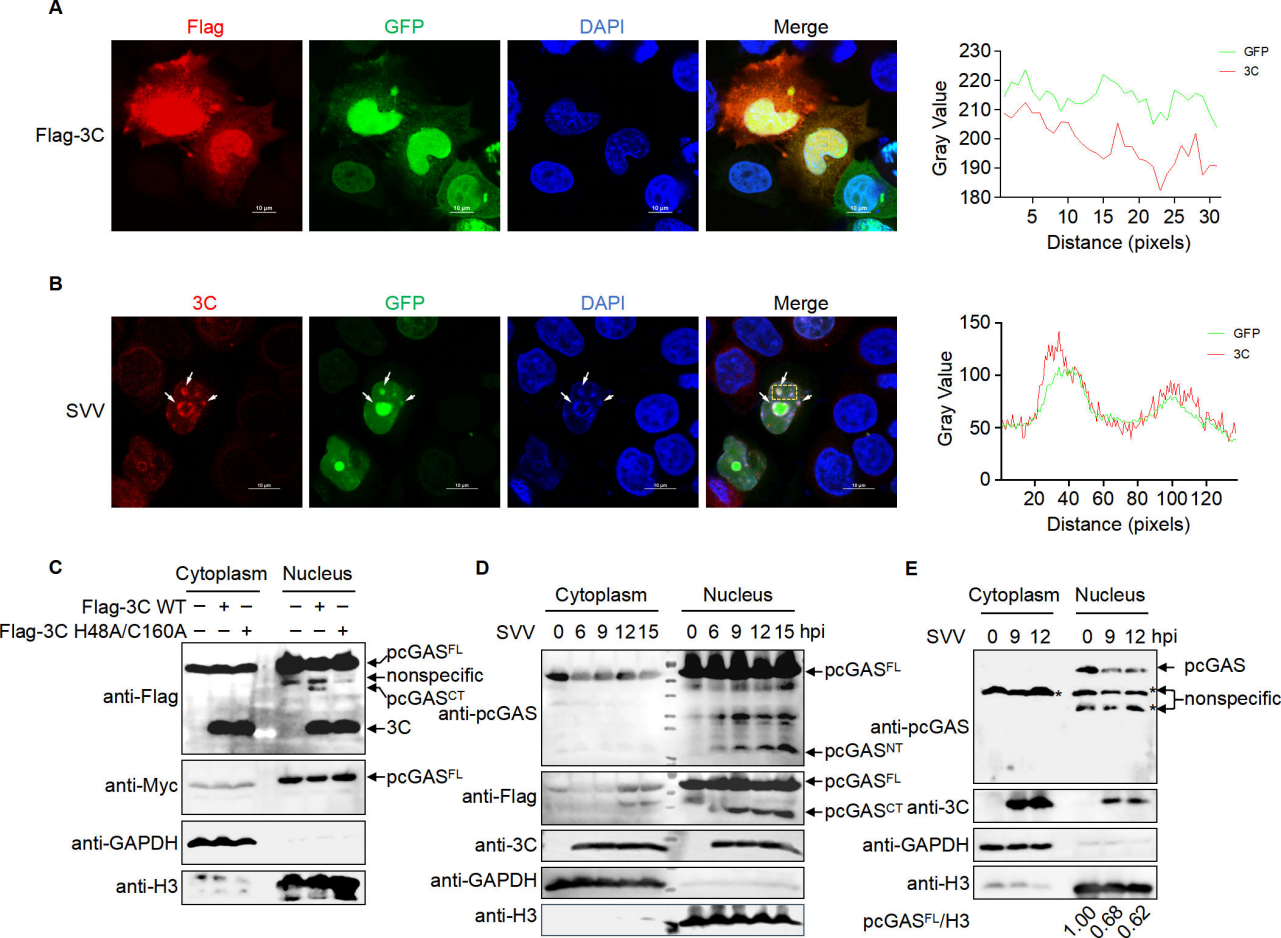
SVV 3C co-localize with and cleaves nuclear cGAS within its N-terminal domain. (**A and B**) Confocal microscopy analysis of co-localization between 3C and pcGAS in the nucleus of HeLa cells stably expressing GFP-pcGAS, either transfected with a plasmid encoding Flag-3C for 18 h (**A**) or infected with SVV (MOI 10) for 12 h (**B**). Scale bars, 10 µm. (**C**) Detection of pcGAS cleavage in the nucleus of HEK-293T cells stably expressing Myc-pcGAS-Flag transfected with a plasmid encoding WT or mutant 3C for 24 h by Western blot analysis. (**D**) Detection of pcGAS cleavage in the nucleus of HEK-293T cells stably expressing Myc-pcGAS-Flag infected with SVV at an MOI of 20 for 0, 6, 9, 12, and 15 h by Western blot analysis. (**E**) Detection of pcGAS expression in the nucleus of PAMs infected with SVV at an MOI of 20 for 0, 9, and 12 h by Western blot analysis. The asterisk (*) in the figure denotes a non-specific band.

To explore whether 3C and pcGAS co-localize in the nucleus, HeLa cells stably expressing GFP-pcGAS-HA were generated using a lentiviral overexpression system and then transfected with Flag-3C-expressing plasmid or infected with SVV. We observed that 3C and pcGAS co-localized in the nucleus during both transfection and infection (Fig. 4A and B). To determine whether 3C cleaves pcGAS in the nucleus, we infected HeLa cells stably expressing GFP-pcGAS-HA with SVV infection and then isolated the nucleus. A cleavage band at ~40 kDa was detected and excised from the SDS-PAGE gel for mass spectrometry analysis. The results showed that the band included both N-terminus and C-terminus of pcGAS (pcGAS^NT^, pcGAS^CT^), likely due to the similar sizes of GFP-pcGAS^NT^ and pcGAS^CT^ (Fig. S8). To confirm these findings, HEK-293T cells stably expressing Myc-pcGAS-Flag were transfected with a plasmid encoding WT or inactive 3C (H48A/C160A). The cleavage bands of pcGAS were detected in the nucleus with WT 3C but not with inactive 3C, indicating that the cleavage of nuclear pcGAS is dependent on the enzymatic activity of 3C (Fig. 4C). Furthermore, we detected both nuclear pcGAS^NT^ and nuclear pcGAS^CT^ in HEK-293T cells stably expressing Myc-pcGAS-Flag at different time points post-infection (Fig. 4D). To verify these findings, porcine alveolar macrophages (PAMs) were infected with SVV. Nuclear components were isolated by lysing the infected PAMs. The endogenous level of pcGAS was significantly reduced in the nucleus upon SVV infection, while cytosolic pcGAS expression was not detected (Fig. 4E).

### Double-stranded DNA significantly enhances the cleavage activity of SVV 3C

**Fig 5:**
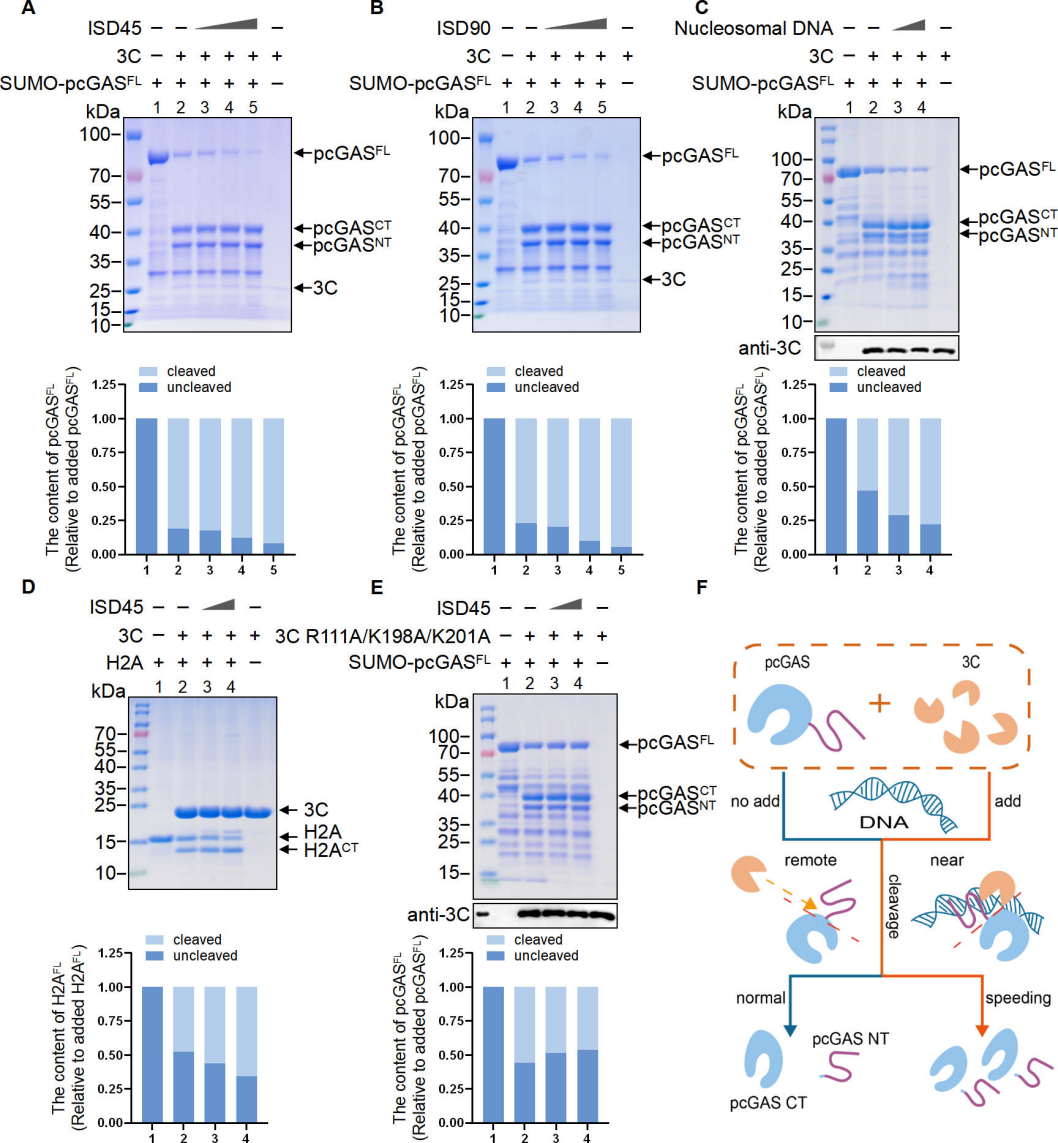
Double-stranded DNA significantly enhances the cleavage activity of SVV 3C. (**A–C**) SDS-PAGE analysis of the cleavage of SUMO-pcGAS^FL^ in reactions containing SUMO-pcGAS^FL^ and 3C, together with ISD45 (**A**), ISD90 (**B**), or nucleosomal DNA (**C**) at increasing DNA concentrations incubated at 37°C for 2 h. The graph in the bottom panel shows the content of pcGAS^FL^. (**D**) SDS-PAGE analysis of H2A cleavage in a reaction containing H2A-6×His and 3C, together with ISD45 incubated at 37°C for 2 h. The graph in the bottom panel shows the content of H2A^FL^. (**E**) SDS-PAGE analysis of the cleavage of SUMO-pcGAS^FL^ in a reaction containing SUMO-pcGAS^FL^ and mutant 3C, together with ISD45 at increasing concentrations incubated at 37°C for 2 h. The graph in the bottom panel shows the content of pcGAS^FL^. (**F**) Schematic diagram illustrating the DNA-enhanced cleavage of pcGAS by 3C protease.

Considering that dimerized cGAS monomers bridge two nucleosome core particles (NCPs) by binding the acidic patch of H2A-H2B and nucleosomal DNA (13, 14), we investigated whether nucleosomal DNA binding to 3C affects the cleavage of nuclear pcGAS. Increasing doses of ISD45, ISD90, or nucleosomal DNA were added to a cell-free system containing SVV 3C and pcGAS recombinant proteins *in vitro*. Surprisingly, we observed increased cleavage of pcGAS by 3C in the presence of ISD45, ISD90, and nucleosomal DNA (Fig. 5A through C). To further explore whether dsDNA enhances 3C’s cleavage activity, different doses of ISD45 were added to a cell-free system containing SVV 3C and H2A recombinant proteins *in vitro*. The results showed that the cleavage of H2A also increased (Fig. 5D). However, when ISD45 was added to a system containing pcGAS and the 3C mutant (R111A/K198A/K201A) with impaired DNA-binding ability, ISD45 no longer promoted the cleavage of pcGAS (Fig. 5E). These results indicate that dsDNA enhances the cleavage activity of 3C, likely due to its binding with 3C, as illustrated in our proposed schematic model (Fig. 5F). Taken together, our results demonstrate that DNA binding to SVV protease 3C significantly enhances the cleavage of pcGAS.

### SVV 3C cleaves nuclear cGAS at the N-terminus to evade immune restriction

**Fig 6:**
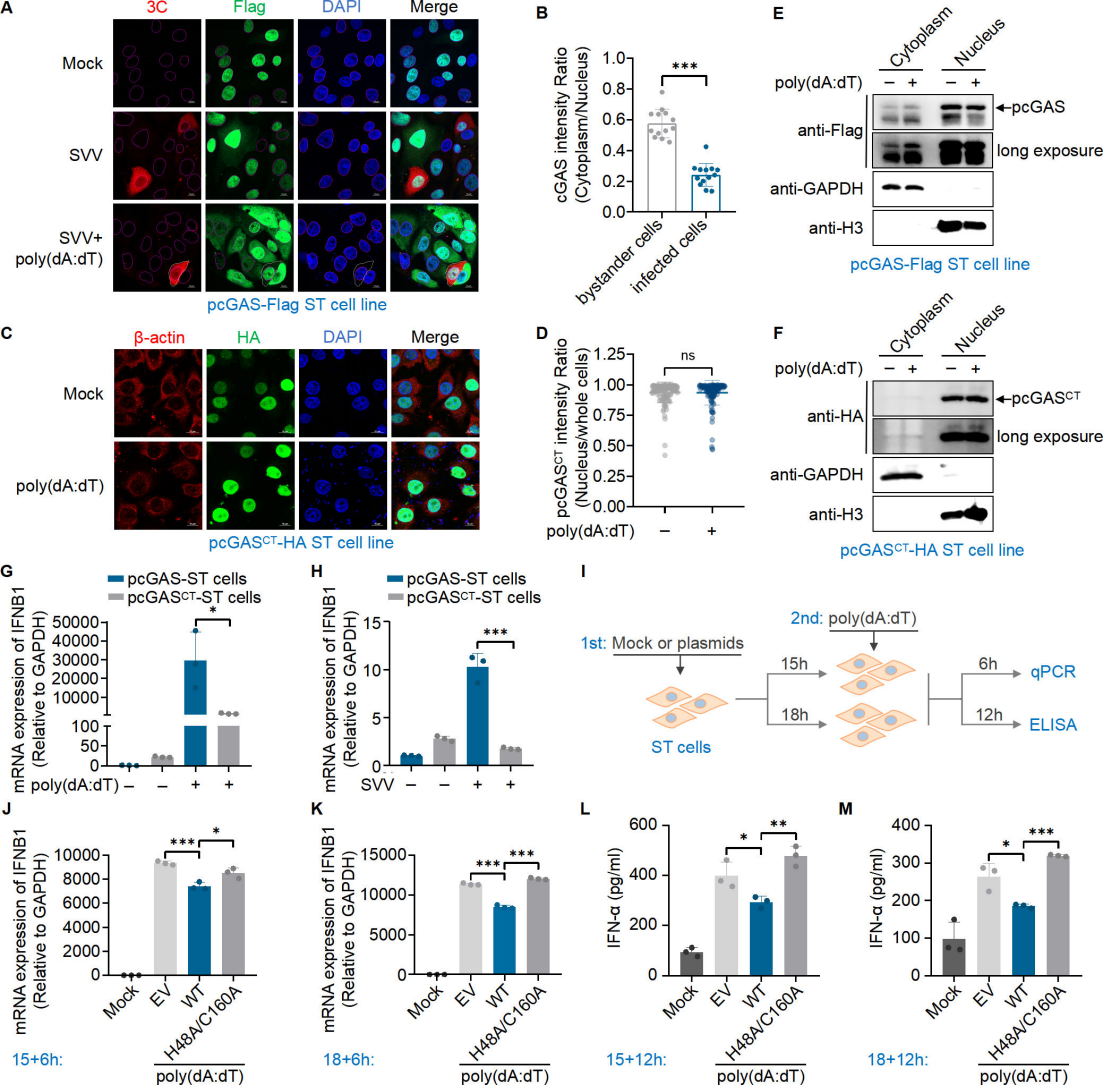
SVV 3C cleaves the N-terminus of nuclear cGAS to evade immune restriction. (**A**) The localization of pcGAS in ST cells stably expressing pcGAS-Flag (pcGAS-Flag-ST cells) treated with or without SVV (MOI 10) for 4 h followed by mock transfection or transfection with poly(dA:dT) for 6 h. Scale bars, 10 µm. (**B**) Relative ratio of pcGAS in the cytoplasm to the nucleus corresponding to (**A**). (**C**) The localization of pcGAS C-terminus in ST cells stably expressing pcGAS^CT(139-495aa)^-HA (pcGAS^CT^-HA-ST cells) following mock-transfection or transfection with poly(dA:dT) for 6 h. Scale bars, 10 µm. (**D**) Relative ratio of pcGAS C-terminus in the nucleus to whole cells corresponding to (**C**). (**E and F**) Western blot analysis of pcGAS in the nucleus and cytoplasm of ST cell line stably expressing the full-length (**E**) or pcGAS C-terminus (139-495 aa) (**F**). (**G**) qRT-PCR analysis of the mRNA level of *IFNB1* (relative to *GAPDH*) in pcGAS-Flag-ST or pcGAS^CT^-HA-ST cells following mock transfection or transfection with poly(dA:dT) for 6 h. (**H**) qRT-PCR analysis of the mRNA level of *IFNB1* (relative to *GAPDH*) in pcGAS-Flag-ST or pcGAS^CT^-HA-ST cells treated with or without SVV (MOI 10) for 9 h. (**I**) Schematic for the co-transfection experiment. (**J and K**) qRT-PCR analysis of the mRNA level of *IFNB1* (relative to *GAPDH*) in ST cells transfected with empty vector or a plasmid encoding WT or mutant 3C for 15 h (**J**) or 18 h (**K**) followed by stimulation with poly(dA:dT) for another 6 h. (**L and M**) Quantification of IFN-α secreted by ST cells transfected with empty vector or a plasmid encoding wild-type or mutant 3C for 15 h (**L**) or 18 h (**M**) followed by stimulation with poly(dA:dT) for another 12 h. IFN-α in culture supernatant was quantified with ELISA. Means ± SD are shown in (**B**) (*n* = 13). Means ± SD are shown in (**D**) (*n* = 124). Means ± SD are shown in (**G, H, J–M**) (*n* = 3). Two-tailed unpaired *t*-test was used for the statistical analysis, *P < 0.1, **P < 0.01, ***P < 0.001. ns, no significance.

Previous studies have demonstrated that nuclear cGAS translocates to the cytoplasm upon DNA stimulation (12, 40, 41). To investigate the translocation of pcGAS, we constructed an ST cell line stably expressing either full length or C-terminus of pcGAS (139-495 aa), referred to as pcGAS-Flag-ST cells or pcGAS^CT^-HA-ST cells, respectively. We stimulated the pcGAS-Flag-ST cell line with poly(dA:dT) for 6 h and then analyzed the subcellular localization of pcGAS-Flag by immunofluorescence. We found that the pcGAS-Flag translocated to the cytoplasm upon poly(dA:dT) stimulation (Fig. S9A). Following SVV infection for 4 h and subsequent stimulation with poly(dA:dT) for 6 h, confocal microscopy analysis indicated that pcGAS-Flag primarily remained in the nucleus in SVV-infected cells (Fig. 6A). Statistical analysis of pcGAS localization in the nucleus or cytoplasm, based on the third row of Figure 6A, showed that the translocation of pcGAS to the cytoplasm was significantly reduced in infected cells compared to bystander cells (Fig. 6B). As a control, pcGAS C-terminus was consistently located in the nucleus in pcGAS^CT^-HA-ST cell lines, regardless of poly(dA:dT) stimulation or SVV infection (Fig. 6C and D; Fig. S9B), which was further validated by Western blot analysis (Fig. 6E and F). We also detected the expression of pcGAS-Flag and pcGAS^CT^-Flag in HEK-293T cells (Fig. S7C).

To assess the impact of the loss of the N-terminal domain on cGAS-mediated immune response, we measured the mRNA expression levels of *IFNB1* in response to poly(dA:dT) stimulation or SVV infection, comparing pcGAS^CT^-HA-ST cells to full-length pcGAS-Flag-ST cells. The results showed significantly higher *IFNB1* mRNA levels in full-length pcGAS-Flag-ST cells compared to pcGAS^CT^-HA-ST cells under both conditions (Fig. 6G and H). To further investigate the effect of 3C on endogenous pcGAS-mediated IFN-β production, ST cells were transfected with either WT 3C or inactive 3C (H48A/C160A) and then stimulated with poly(dA:dT) (Fig. 6I). Following poly(dA:dT) stimulation, both the *IFNB1* mRNA levels and IFN-α secretion were reduced in ST cells expressing WT 3C but not in those expressing the inactive 3C (H48A/C160A) (Fig. 6J through M). As the 1-20 amino acid region of 3C is crucial for its nuclear localization (Fig. 1K), we hypothesized that a 3C mutant lacking this region would induce a stronger immune response. Attempts to rescue an SVV virus lacking the first 20 amino acids of 3C were unsuccessful, likely due to conformational changes that inactivated the protein. To test this hypothesis, we predicted the protein structure of 3C ∆1-20 and compared it to the WT 3C using AlphaFold3. The analysis revealed that the secondary structure near the enzyme’s active site in 3C ∆1-20 changed from a β-sheet to a random coil (Fig. S10A), which may impair its enzymatic activity. In our previous study, we found that SVV 3C cleaves pcGAS through its protease activity. To determine whether the deletion of amino acids 1-20 affects the enzymatic activity of 3C, we transfected HEK-293T cells with a plasmid encoding Myc-pcGAS-Flag, along with plasmids expressing either WT Flag-3C or Flag-3C (∆1-20 aa). Western blot analysis showed that the 3C (∆1-20 aa) mutant lost its ability to cleave pcGAS (Fig. S10B). In addition, we transfected ST cells with plasmids encoding either WT 3C or the 3C (∆1-20 aa) mutant, followed by stimulation with poly(dA:dT) for 15 h. Samples were collected after 6 h for qRT-PCR. The results showed that, unlike WT 3C, the 3C (∆1-20 aa) mutant failed to inhibit poly(dA:dT)-induced IFN-β expression (Fig. S10C). These findings indicate that the deletion of the first 20 amino acids induces conformational changes that disrupt the key enzymatic residues of 3C, thereby abolishing its inhibitory function. In conclusion, the N-terminal domain of nuclear pcGAS is essential for IFN production, and SVV protease 3C suppresses type I IFN by cleaving this domain, preventing the translocation of nuclear pcGAS to the cytoplasm.

## DISCUSSION

The immune system plays a crucial role in defending against pathogenic attacks to maintain cellular homeostasis. The mitochondria and nucleus are key organelles essential for cell survival, growth, and division (42). It is widely reported that viral infections cause mtDNA to leak into the cytoplasm. However, reports of nuclear DNA leakage into the cytoplasm upon infection, which serves as a danger signal, are rare. For example, SARS-CoV-2 infection induces syncytia formation, leading to the translocation of chromatin and micronuclei from the nucleus to the cytoplasm (43, 44). Similarly, Noroviruses NS4 has been shown to facilitate nuclear DNA leakage (35). Consistent with these findings, we found that SVV 3C translocates into the nucleus and induces nuclear DNA leakage. Furthermore, SVV 3C cleaves histone H2A and disrupts nucleosome function, which may contribute to nuclear DNA leakage. Many studies have reported that 3C proteases of picornaviruses possess the capability to translocate into the nucleus (30, 31, 33); however, whether their functions are similar to those of SVV 3C remains to be explored. To identify the key residues required for 3C translocation into the nucleus, we constructed deletions of 3C. We found that the expression of 3C ∆1-20 was significantly reduced in the nucleus (Fig. 1K), suggesting that amino acids 1-20 are crucial for its nuclear translocation. Recent reports indicate that SVV 3C binds to phospholipid (45), raising the intriguing possibility that phospholipid binding and the enzymatic activity of 3C may play a role in facilitating its nuclear translocation.

cGAS was initially recognized as a cytoplasmic double-stranded DNA sensor crucial for IFN-I induction. However, emerging evidence suggests that cGAS is predominantly located in the nucleus (16, 40, 46), a finding consistent with our results. Numerous studies have demonstrated viral strategies for evading and degrading cGAS to counteract innate immune recognition. For example, the Dengue virus protease NS2B targets cGAS for lysosomal degradation (23), and its protease NS2B3 cleaves cGAS (47). Additionally, Zika virus NS1 recruits USP8 to stabilize caspase-1, which then cleaves cGAS. Human cytomegalovirus (HCMV) UL31 competes with cGAS for DNA binding (27), and Kaposi’s sarcoma-associated herpesvirus (KSHV) ORF52 interacts with both DNA and cGAS to inhibit the enzymatic activity of cGAS (26). To date, there is no report of viral strategies specifically targeting nuclear cGAS during infection. We discovered that SVV protease 3C can cleave nuclear cGAS within the N-terminal domain. Interestingly, ISD45/ISD90 and nucleosomal DNA significantly enhanced the cleavage of pcGAS and H2A by SVV 3C in a cell-free system. We hypothesized that this phenomenon arises from conformational changes in 3C induced by its association with DNA, or from the proximity effect facilitated by the substrates’ interaction with DNA. Our study has demonstrated that SVV 3C possesses DNA-binding ability, similar to pcGAS and H2A. Thus, we propose that this enhancement is likely mediated by the proximity of substrates facilitated by the interaction of SVV 3C and dsDNA. Moreover, we previously reported that the binding of DNA to NLRC3 enhances its ATPase activity 10-fold through conformational changes (48). Similarly, the binding of DNA to SVV 3C may induce conformational changes that enhance its protease activity.

**Fig 7:**
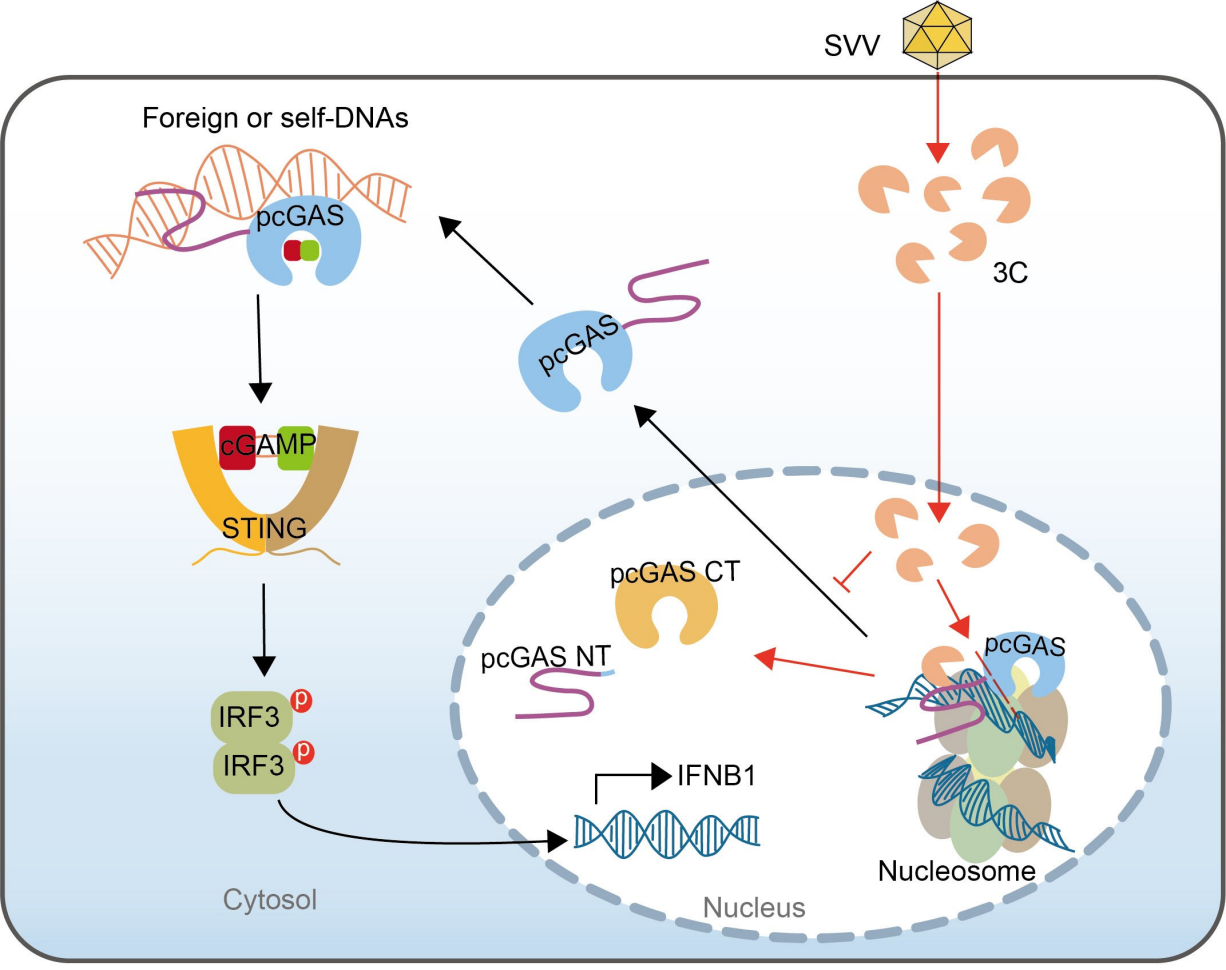
Schematic model of SVV protease 3C-mediated cleavage of nuclear pcGAS to attenuate type I interferon. SVV infection results in the translocation of 3C proteins into the nucleus, where they bind to nucleosomal DNA. Concurrently, 3C cleaves nuclear cGAS, impairing its export and inhibiting the immune response.

The B site of nuclear cGAS interacts with nucleosomes, leading to the inhibition of cGAS, which underscores the role of nuclear cGAS in maintaining the genomic stability (13, 15, 17). Additionally, the association of HIV DNA with nuclear cGAS is crucial for antiviral defense (19). Previous reports confirmed the translocation of nuclear cGAS to the cytoplasm upon poly(dA:dT) stimulation (12, 40, 41) and further revealed that the N-terminal domain of cGAS predominantly localizes in the cytoplasm, while the C-terminal domain mainly resides in the nucleus (22, 40). Furthermore, we showed that pcGAS lacking its N-terminal domain did not exhibit translocation from the nucleus to the cytosol upon poly(dA:dT) stimulation, highlighting the crucial role of the cGAS N-terminal domain in cytoplasmic localization. Proper cytoplasmic localization is essential for cGAS to detect cytoplasmic DNA and trigger IFN responses (21, 25). We observed that SVV 3C proteases translocate into the nucleus and cleave the nuclear cGAS N-terminus, preventing its translocation to the cytoplasm and thereby evade immune restriction (Fig. 7). Although leaked DNA in the cytosol could potentially activate cGAS, the impaired translocation of nuclear cGAS likely leads to a failure in DNA recognition, a phenomenon that warrants further investigation.

## MATERIALS AND METHODS

### Cells and viruses

HEK-293T cells, ST cells, PK-15 cells, PAMs, and HeLa cells were cultured in Dulbecco’s Modified Eagle Medium (DMEM) (MACGENE, CM10013) or Roswell Park Memorial Institute (RPMI) 1640 Medium (MACGENE, CM10040) containing 10% fetal bovine serum (FBS) (PlantChemMed, PC-00001) at 37°C with 5% CO_2_, and the medium culturing PAMs was added with 0.1 mM non-essential amino acid (NEAA). HEK-293T and HeLa cell lines stably expressing pcGAS were constructed using a lentivirus overexpression system. SVV and EMCV were stored in our laboratory. Cell images were analyzed using Falcon S400, Intelligent cell imaging and analysis system (Alicelligent Technologies).

### Antibodies

The following antibodies were used in this study. Histone H3 (D1H2) XP rabbit monoclonal antibody (MAb) (4499), rabbit anti-Myc MAb (2278), anti-mouse IgG horseradish peroxidase (HRP)-linked antibody, and anti-rabbit IgG HRP-linked antibody were purchased from Cell Signaling Technology (Beverly, MA, USA). Anti-HA antibody produced in rabbit (H6908), anti-DNA MAb (CBL186), and mouse anti-Flag MAb (F1804) were obtained from Sigma-Aldrich (St. Louis, MO, USA). Rabbit anti-GAPDH polyclonal antibody (PAb) (10494-1-AP) was purchased from Proteintech Group (Chicago, IL, USA). Mouse anti-β-Actin antibody (C4) (sc-47778) was purchased from Santa Cruz Biotechnology. Alexa Flour 633 goat anti-mouse IgG (H+L) (A21052) and Alexa Flour 488 goat anti-rabbit IgG (H+L) (A11034) were obtained from Thermo Fisher Scientific (Waltham, MA, USA). Mouse anti-dsRNA MAb J2 (10010500) was from Scicons. Mouse anti-SVV 3C polyclonal antibody (PAb) and mouse anti-pcGAS PAb were prepared by our laboratory.

### Plasmids

SVV, EV-A71, EMCV, CVB3, PV, and HRV 3C plasmids were preserved in the laboratory. The N-terminal GFP/C-terminal hemagglutinin (HA) or the N-terminal Myc/C-terminal Flag double-tagged cGAS was ligated to pNL vector at the sites of XhoI and NheI in frame. The cGAS was ligated to His6-SUMO-pET28a vector at the sites of BamHI and HindIII. N-terminal Flag-tagged 3C proteases of picornaviruses were ligated to pcDNA3.1 or 3 × Flag-CMV-10 vector. The 3C protease of SVV was ligated to His_6_-SUMO-pET28a vector. C-terminal HA-tagged H2A, H2B, H3, and H4 were ligated to pcDNA3.1. Mutant and truncated plasmids of SVV 3C were ligated to pcDNA3.1.

### Protein expression and purification

SVV 3C, EV-A71 3C, and EMCV 3C were cloned into the His_6_-SUMO-pET28a vector, and porcine histone H2A (Gene ID: 110261666), H2B (Gene ID: 110261663), H3 (Gene ID: 100156741), and H4 (Gene ID: 110261487) were cloned into pET21a vector, followed by transformation into *E. coli* BL21 (DE3) (TransGen Biotech, CD601). The bacteria were cultured in Luria-Bertani (LB) medium with appropriate kanamycin. When OD600 reached at 1.0, the proteins were induced overnight at 16°C with 1 mM isopropyl β-D-1-thiogalactopyranoside (IPTG, 18070). The bacteria were lysed in a buffer containing 50 mM Tris-HCl (pH 8.0) and 300 mM NaCl. The viral proteins were purified using the previously established method in our laboratory, while histone purification was conducted following the protocol described by Michalski et al. (14).

### Detection of the cleavage of pcGAS in nucleus

The cells were plated in a 6 cm dish. When the cells grew to an appropriate confluence, they were infected with SVV (multiplicity of infection [MOI] 20) or transfected with the WT 3C or mutant 3C plasmids. The infected cells were collected at different time points, and the transfected cells were collected at 20 h post-transfection. The samples were collected and separated by Nuclear and Cytoplasmic Extraction Reagents (Thermo Scientific, 78835). After protein denaturation, Western blot analysis detection was performed.

### *In vitro* cleavage assay and mass spectrometry

The target protein and SVV 3C recombinant protein were incubated for 2 h at 37°C in the reaction buffer, and then the protein was analyzed by SDS-PAGE to detect the formation of cleavage bands. The cleavage bands were treated with trypsin and then detected by mass spectrometry. The results were compared and analyzed by Matrix Science. The reaction buffer contained 50 mM HEPES (pH 7.5), 3 mM EDTA, 150 mM NaCl, 0.005% (vol/vol) Tween-20, and 10 mM dithiothreitol (DTT). The 25 µL reaction system contains 20 µg of histone protein and 1, 5, 10, 15, or 20 µg of 3C. In the cleavage reaction system between nucleosomes and 3C protein, 10 µL of nucleosomes and 3C protein (0.5, 1, 5, 10, 15, or 20 µg, respectively) were incubated. Besides, the 80, 160, or 320 µM ISD45/ISD90 was added to the reaction system containing 15 µg of pcGAS and 0.2 µg of 3C protease.

### The detection of nuclear DNA in the cytoplasm

Samples were collected from cells that were infected with viruses or transfected with plasmids at different time points. One-quarter of the sample was extracted for total mRNA and reverse transcribed into cDNA, and the remaining three-quarters were processed using Nuclear and Cytoplasmic Extraction Reagents to obtain cytoplasmic components, which were then treated with QIAamp DNA Mini and Blood Mini kit (QIAGEN, 51306) to obtain the cytoplasmic DNA. Cytoplasmic DNA and cDNA were taken from each sample, and the GAPDH of the corresponding sample was used as an internal reference to detect nuclear DNA by qRT-PCR. The primers used for nuclear DNA detection are as follows: 18S-F: TAGAGGGACAAGTGGCGTTC, 18S-R: CGCTGAGCCAGTCAGTGT, Myc-F: AAGGACTATCCTGCTGCCAA, Myc-R: CCTCTTGACATTCTCCTCGG, nuclear DNA1-F: GTTCCTGGCTCTGGATCTTGG, nuclear DNA1-R: GCCACTGCCTCGCATGA.

### Immunofluorescence

The tablets were placed in a 24-well plate, and when the cells reached the appropriate confluence, they were transfected with plasmids or infected with viruses. Cell samples were collected at different time points. The supernatant was discarded, and cells were washed with phosphate-buffered saline (PBS) once. Next, 4% paraformaldehyde was added and incubated at room temperature for 15 min or 4°C overnight. The paraformaldehyde was discarded and washed with PBS three times, and the plate was gently shaken for 3 min each time. The cells were treated with 1% Triton X-100 at room temperature for 10 min and then washed three times with PBS. Five percent bovine serum albumin (BSA) (Sigma-Aldrich, A7906-100G) was incubated at room temperature for 30 min. Then, cells were incubated with corresponding primary antibody at room temperature for 2 h or 4°C overnight and washed with PBS three times for 5 min each time, followed by incubation with Alexa Flour 633 goat anti-mouse IgG (H+L) antibody or Alexa Flour 488 goat anti-rabbit IgG (H+L) antibody for 30 min. Then, cells were treated with 0.1 µg/mL 4′,6-diamidino-2-phenylindole (DAPI) (Beyotime, C1002) for 5 min. After washing with PBS, the stained cells were observed using a Nikon A1 confocal microscope. Images were collected and analyzed using NIS-Elements AR.

### Nucleosome extraction and ESC digestion

The nucleosomes were extracted using Nucleosome Preparation Kit (Active Motif, 53504) after collecting infected or mock-treated ST cells in a 10 cm dish. The cells were first lysed for 30~60 min. Following centrifugation at 5,000 rpm at 4°C for 10 min, the supernatant was discarded, and the pellet was resuspended in digestion buffer and treated with ESC at 37°C for various time points. Digestion was immediately terminated by adding 0.5 M EDTA and incubating on ice for 10 min. A portion of the ESC-digested samples was used to detect the histone H3 content by Western blot, ensuring consistent loading amounts between infected and mock nucleosomes samples. The remaining samples were either stored at −80°C or directly treated with RNase at 37°C for 1 h, followed by digestion with protease K at 45°C for 2 h. Based on the Western blot results, some samples were analyzed using agarose gel electrophoresis to evaluate the digestion of nucleosomes by ESC.

### Electrophoretic mobility shift assays (EMSA)

Nucleosomes were extracted from untreated cells and digested with ESC for 15 min to maximize mononucleosomes yield. Different doses of 3C protein and nucleosomes were incubated for 30 min in reaction buffer and were blended on ice. ISD45 or HSV60 (1 µg) was incubated with 3C protease at molar concentration ratios of 1:0, 1:2, 1:4, 1:8, 1:16, and 1:32 and was blended on ice. Similarly, 1 µg of nucleosomal DNA was incubated with varying doses of 3C protein (0, 5, 10, 15, and 20 µg). The mixtures were resolved on a 1% agarose gel using an electrophoresis buffer of 40 mM Tris-HCl (pH 9.2) at a constant voltage of 100 V.

### Isothermal titration calorimetry (ITC)

Before ITC measurements, the buffers of all samples were replaced with PBS. ITC data were collected using a MicroCal ITC 200 titration microcalorimeter at 25°C. In a typical experiment, a total of 19 injections (each of 2 µL) of the SVV 3C sample at 100 µM were injected into a 300 µL of ISD45 solution at 10 µM. The raw ITC data were processed using Origin 7.0 software (MicroCal), and the curves were fitted to a single-site binding model.

### Protein-DNA structural modeling

To obtain a molecular model of SVV 3C binding to DNA, the 3C (PDB: 8GPH) and DNA (PDB: 5FKW) structures were analyzed using HADDOCK2.4 on the website (https://wenmr.science.uu.nl/haddock2.4/), following the methodology described by van Zundert et al. and Honorato et al. (49, 50).

### Nucleus and cytoplasm extraction

Cells were harvested at appropriate time points after infection of viruses or transfection with plasmids. According to the Nuclear and Cytoplasmic Extraction Reagents (Thermo Scientific, 78835), the cells were lysed for 30~50 min and vortexed every 10 min, then the CERII reagent was added for 5~10 min. Moreover, the supernatant containing cytoplasmic components was obtained by centrifugation at 16,000 × *g*. The NER reagent was added to insoluble (pellet) fraction, and ultrasound was performed using a 25% power ultrasound instrument until the liquid became clear. The nuclear components were obtained from supernatant by centrifugation of 16,000 × *g*.

### Stable expression cell lines construction

The lentivirus packaging system was used to construct stable expression cell lines. First, the target fragment was ligated into pNL vector, and then the pNL plasmid containing the target gene was transfected into HEK-293T cells, together with Pakage and VSV-G plasmids. After 48 h, the supernatant containing the virus was collected and filtered to infect target cells. The expression of the target protein was detected by immunofluorescence or Western blot.

### ChIP-qPCR

ST cells were uniformly cultured in a 10 cm dish and infected with SVV (MOI 20). After overgrowing, the cells were digested with 0.25% trypsin, and the digestion was terminated with 9 mL of culture medium. Additionally, the cells were collected in 15 mL centrifuge tube. Two hundred forty-three microliters of 37% formaldehyde was added to a final concentration of 1%. After mixing, it was cross-linked in an incubator at 37°C for 10 min, and then glycine was added to a final concentration of 0.125 M at room temperature for 5 min to terminate the cross-linking. After centrifugation at 2,000 rpm for 3 min, the supernatant was discarded, and the cells were rinsed with PBS three times. Then, the samples were lysed for 5 min. Nucleosomes were broken down by ultrasound to a length between 100 and 500 bp. The complex was obtained by co-immunoprecipitation assay, digested with protease K for 2 h, and de-crosslinked at 65°C overnight. Then, the supernatant was collected, and DNA was extracted using QIAamp DNA Mini and Blood Mini kit (QIAGEN, 51306). Finally, the CT value of the target DNA in the supernatant was detected by qPCR, and the copy number of the target gene was calculated by establishing a standard curve. Primers for the detection: nuclear DNA1: F: GTTCCTGGCTCTGGATCTTGG, R: GCCACTGCCTCGCATGA, nuclear DNA2: F: GGAGAGGTGTGGTAGCGTTG, R: GGTTTCTGGAAGAGGGGATG.

### qRT-PCR

To detect the mRNA level of IFN-β in ST cells, ST cells were transfected with an empty vector or plasmids expressing WT or double-mutant 3C for 15 or 18 h, followed by transfection with poly(dA:dT) for an additional 6 h. The mRNA level of IFN-β in ST cells was quantified by quantitative real-time PCR (qRT-PCR). Briefly, RNA was extracted from whole-cell lysates using the RNAsimple Total RNA Kit (TIANGEN, DP419) according to the manufacturer’s instructions. The RNA was reversely transcribed into cDNA using HiScript II Q RT SuperMix for qPCR (+gDNA wiper) Kit (Vazyme, R223-01), and the cDNAs were subjected to amplification using gene-specific primers and ChamQ SYBR qPCR Master Mix (Vazyme, Q712-02). Amplification of GAPDH served as an internal control, and *IFNB1* expression levels were calculated using the 2^-ΔΔCT^ method.

### Enzyme-linked immunosorbent assay (ELISA)

IFN-α concentrations were determined using a sandwich ELISA following the manufacturer’s instructions for the Porcine IFN-alpha ELISA Kit (Invitrogen). Briefly, standards and samples were added into wells precoated with IFN-α antibody and incubated for 2.5 h at room temperature with gentle shaking. After the wells were washed four times to washing buffer, biotin-conjugated IFN-α antibody was added to each well for incubation for 1 h. The wells were washed four times, and streptavidin-HRP solution was added to each well for 45 min. After the wells were washed, TMB Substrate was added to the wells for 15 min. The reaction was stopped by adding Stop Solution. The absorbance plate was read at 450 nm using a Microplate Reader.

### Quantification and statistical analysis

All the graphs and relevant statistical tests used in the work were created by GraphPad Prism version 8.0.1. Data were expressed as mean ± standard deviation (SD) and were statistically analyzed with a two-tailed unpaired Student’s *t*-test. A *P* value of < 0.05 was considered to be statistically significant. *P < 0.05; **P < 0.01; ***P < 0.001; ns, no significance.

## SUPPLEMENTAL MATERIAL

Supplemental material mbio.03395-24-s0001.docx Fig. S1 to S10; Table S1.

ASM does not own the copyrights to Supplemental Material that may be linked to, or accessed through, an article. The authors have granted ASM a non-exclusive, world-wide license to publish the Supplemental Material files. Please contact the corresponding author directly for reuse.
