# Supplementary material for: Viral protease binds to nucleosomal DNA and cleaves nuclear cGAS that attenuates type I interferon
Source: mBio. 2025 Feb 25;16(4):e03395-24. doi: 10.1128/mbio.03395-24 (PMC11980361; doi:10.1128/mbio.03395-24)
Supplement: Supplemental material — Fig. S1 to S10; Table S1. [file mbio.03395-24-s0001.docx]

**Figure S1. SVV infection induces nuclear DNA leakage.**


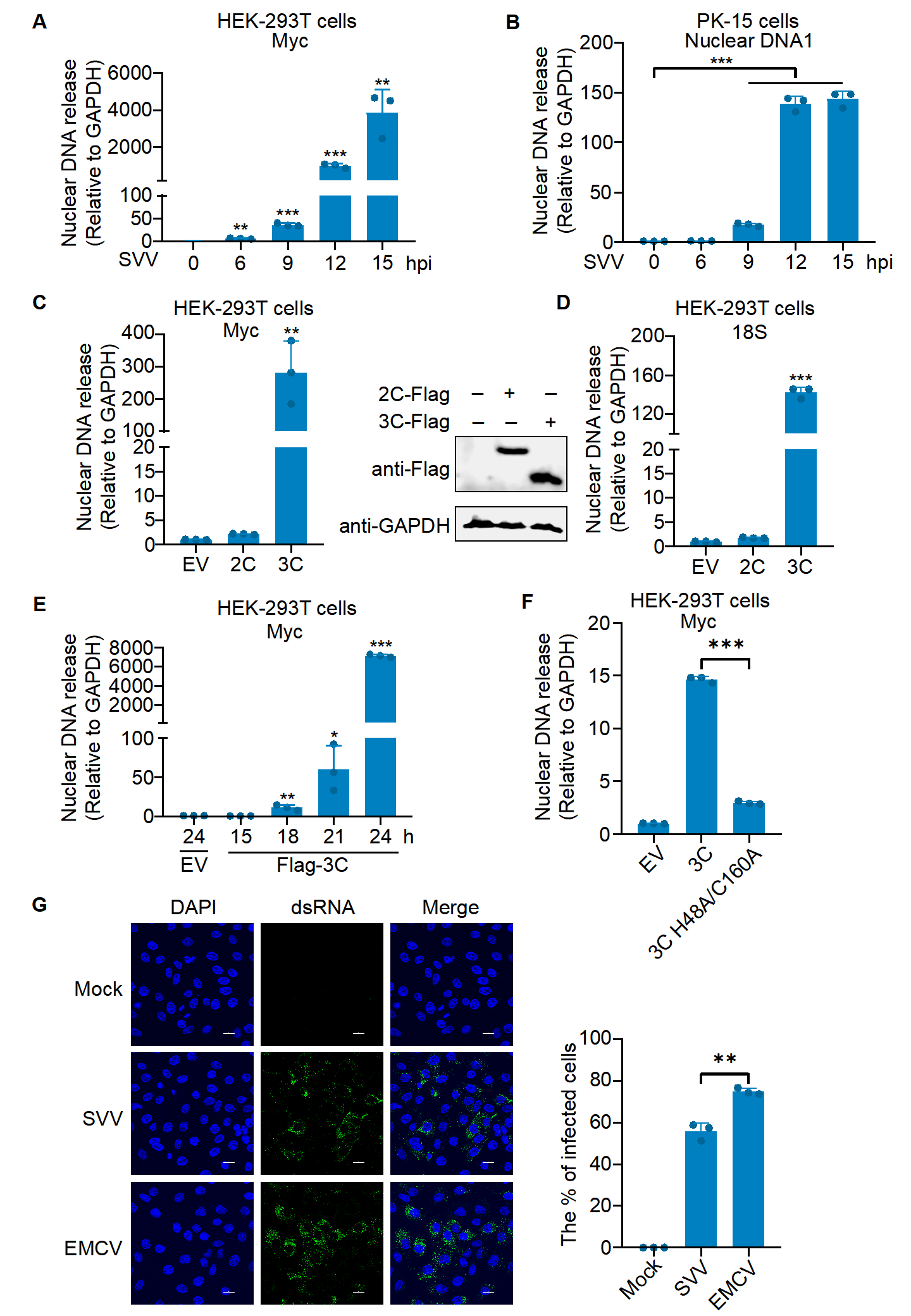


**A**, qPCR analysis of Myc in the cytoplasm of HEK-293T cells infected with SVV at MOI of 10 for 0, 6, 9, 12, 15 or 18 h. **B**, qPCR analysis of nuclear DNA in the cytoplasm of PK-15 cells infected with SVV at MOI of 10 for 0, 6, 9, 12, 15 or 18 h. **C**-**D**, qPCR analysis of 18S (**C**) and Myc (**D**) in the cytoplasm of HEK-293T cells mock-transfected or transfected with a plasmid expressing Flag-3C or Flag-2C for 24 h and western blot analysis of expression of 3C and 2C. **E**, qPCR analysis of Myc in the cytoplasm of HEK-293T cells mock-transfected or transfected with a plasmid expressing Flag-3C for 15, 18, 21, or 24 h. **F**, qPCR analysis of Myc in the cytoplasm of HEK-293T cells transfected with a plasmid expressing WT or mutant 3C for 21 h. **G**, Confocal microscopy analysis of dsRNA in mock ST cells or ST cells infected with SVV or EMCV at MOI of 10 for 6 h. The graph in the right panel showed the quantified percentage of infected cells. Results are representative of three biological replicates. Means ± SD are shown in **A**-**G** (n = 3). Two-tailed unpaired t-test was used for the statistical analysis, *P < 0.1, **P < 0.01, ***P < 0.001.

**Figure S2. Picornavirus protease 3C translocates to the nucleus during** **transfection and infection.**


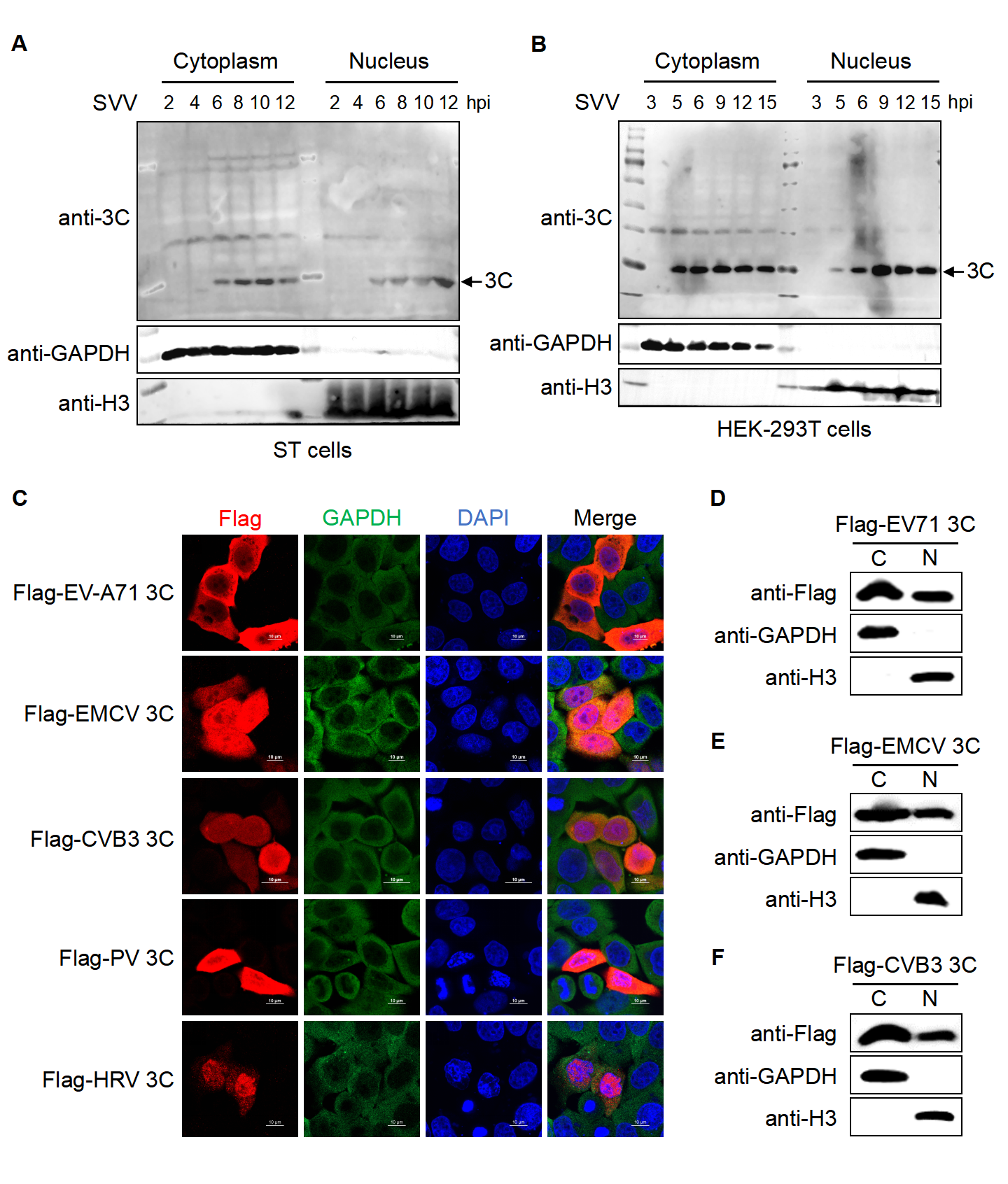


**A-B**, Western blot analysis of 3C in the nucleus and cytoplasm of ST or HEK-293T cells infected with SVV at MOI of 10. **C**, Confocal microscopy analysis of 3C localization in HeLa cells transfected with a plasmid encoding 3C from EV-A71, EMCV, CVB3-28, Human poliovirus 3 strain Sabin 3 or HRV-A16 for 18 h. Scale bars, 10 µm. **D-F**, Western blot analysis of 3C in the nucleus and cytoplasm of HeLa cells transfected with a plasmid encoding EV-A71 3C (**D**), EMCV 3C (**E**) or CVB3 3C (**F**) corresponding to (**C**).

**Figure S3. The N-terminus of 3C dictates its translocation to the nucleus.**


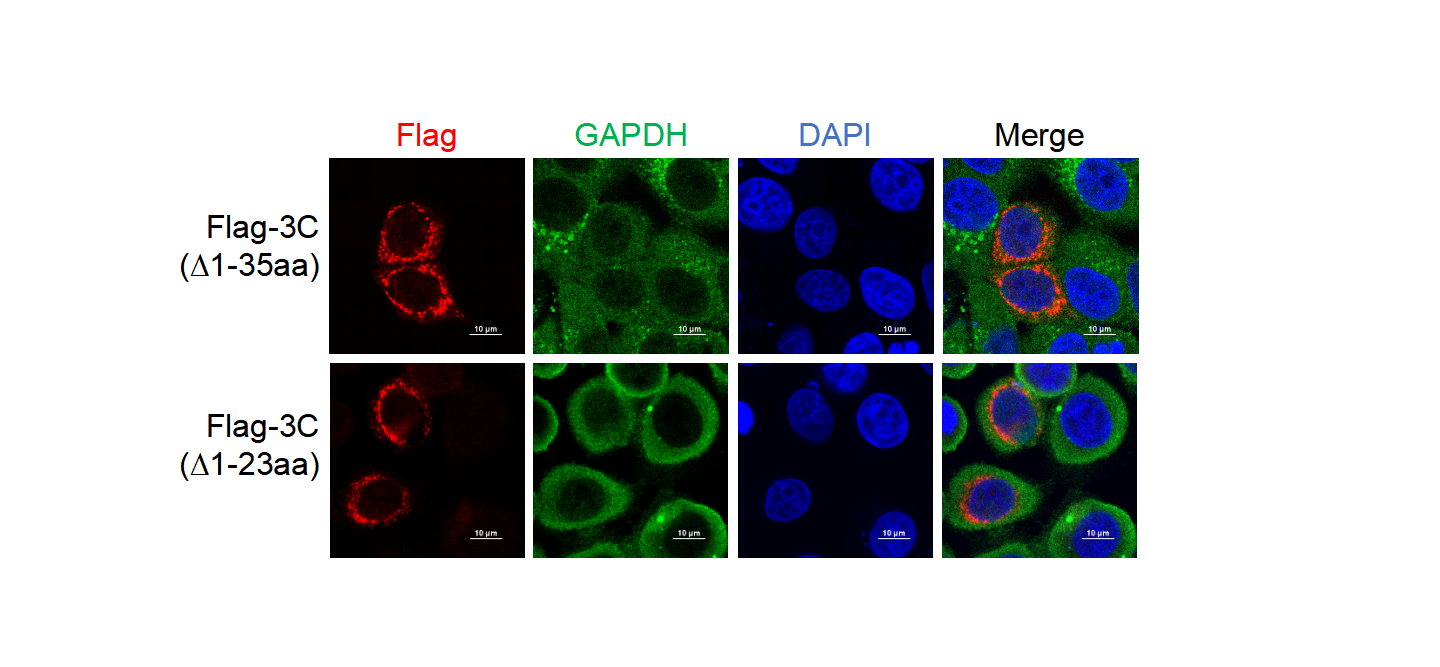


Confocal microscopy analysis of 3C localization in HeLa cells transfected with plasmids encoding different 3C mutants. Scale bars, 10 µm.

**Figure S4. Multiple sequence alignment of H2A from various species.**


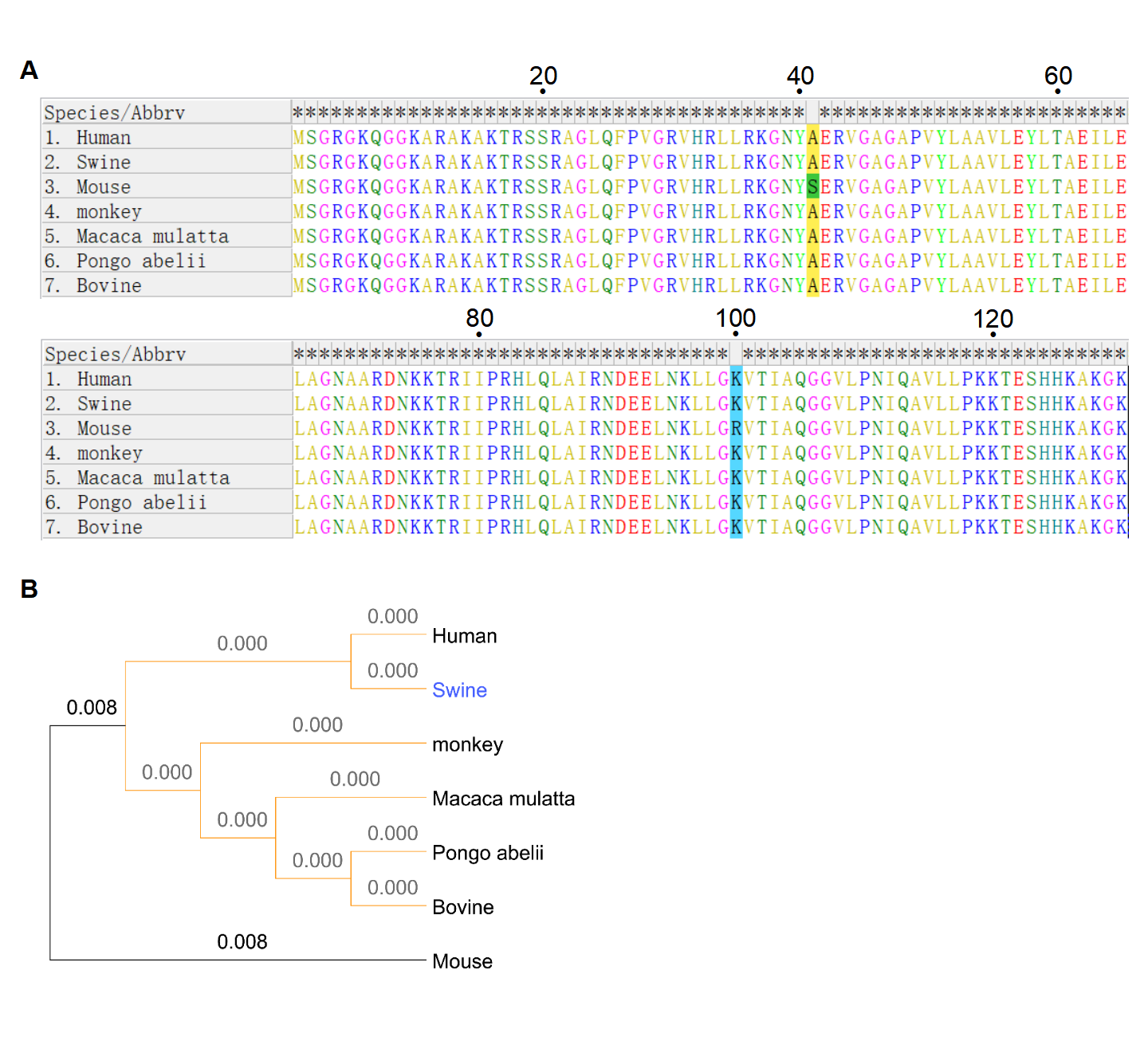


**A**, Multiple sequence alignment of H2A from various species. **B**, The phylogeny and multiple sequence alignment analysis of H2A from different species.

**Figure S5. SVV protease 3C impairs nucleosome function.**


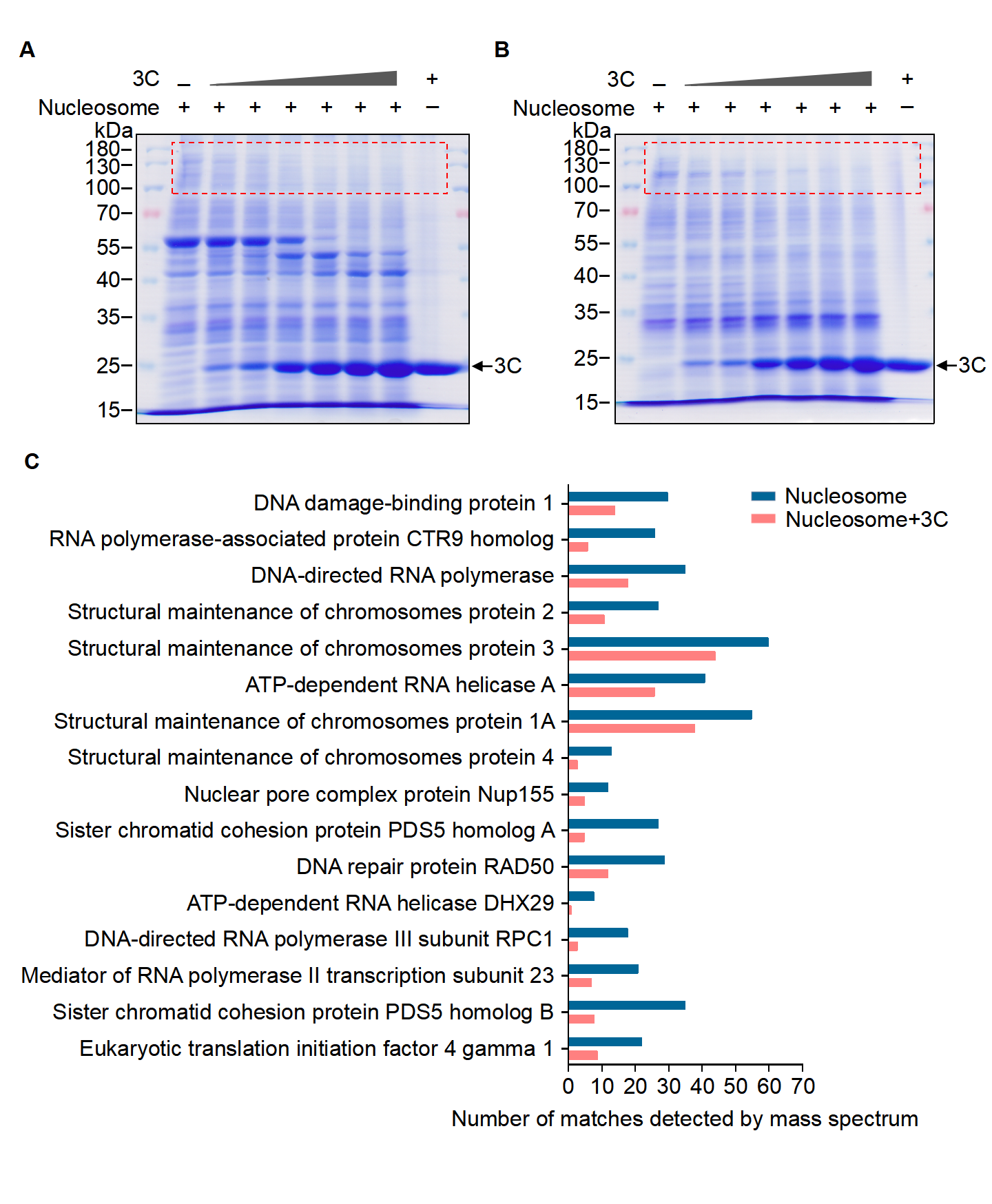


**A**-**B**, SDS-PAGE analysis of incubation system of recombinant protein 3C and nucleosomes extracted from ST cells (**A**) or HEK-293T cells (**B**) for 2 h at 37 °C. **C**, Detection of the effect of 3C protein on the function of nucleosomes. Mass spectrometry analysis of the bands between 100 and180 kDa in SDS-PAGE gel of (**B**).

**Figure S6. SVV 3C inhibits the digestion of DNA by ESC.**


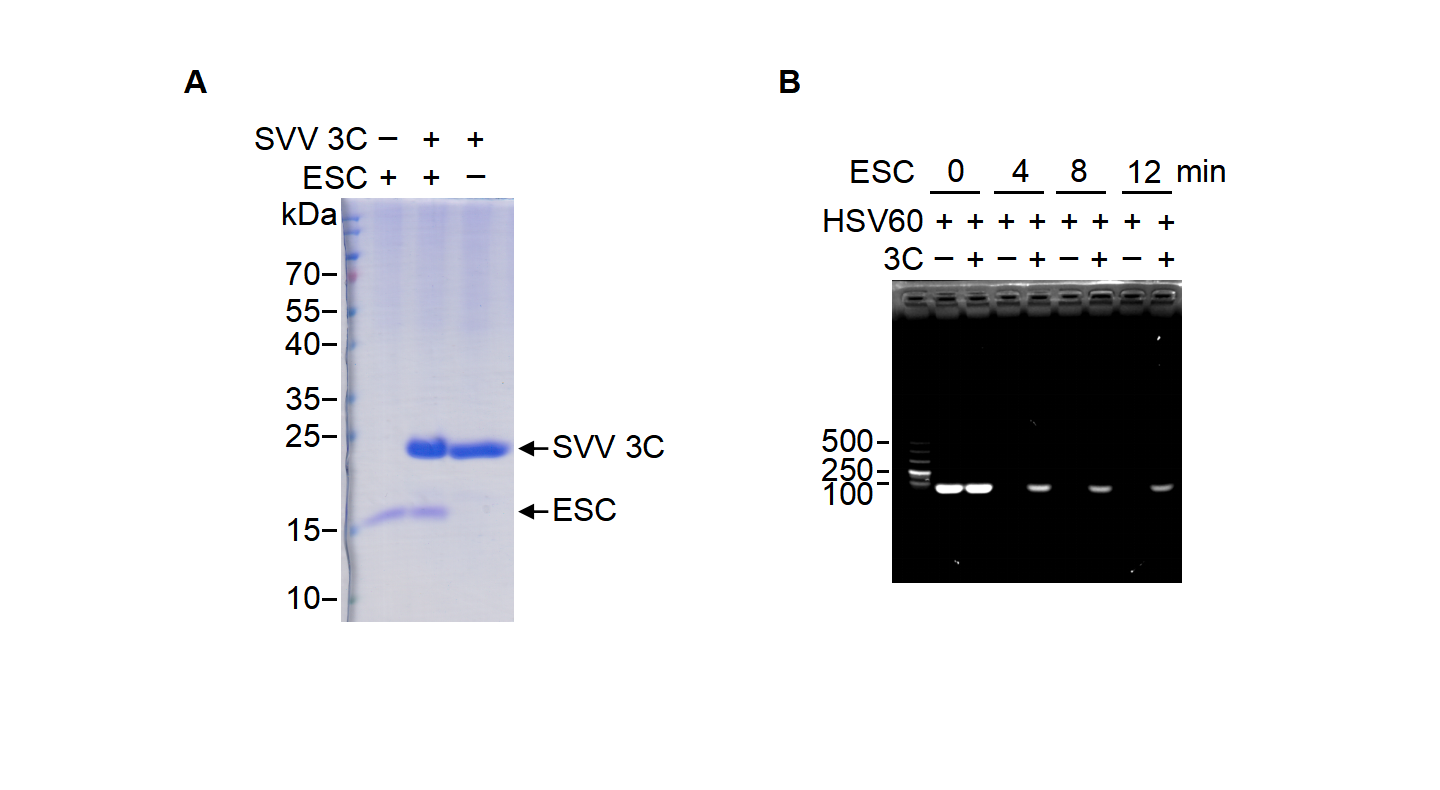


**A**, SDS-PAGE analysis of the effect of 3C on ESC in a reaction containing 15 μl of ESC and 2.5 μg of 3C protein at 37 °C for 2 h. **B**, HSV60 and recombinant protein 3C were mixed at a molar ratio of 1:16 and incubated with ESC at 37 °C for 4, 8, and 12 min, respectively. The reaction was stopped by adding EDTA, followed by a 2-hour treatment with protease K before agarose gel electrophoresis.

**Figure S7. The mutant SVV 3C recombinant proteins are purified.**


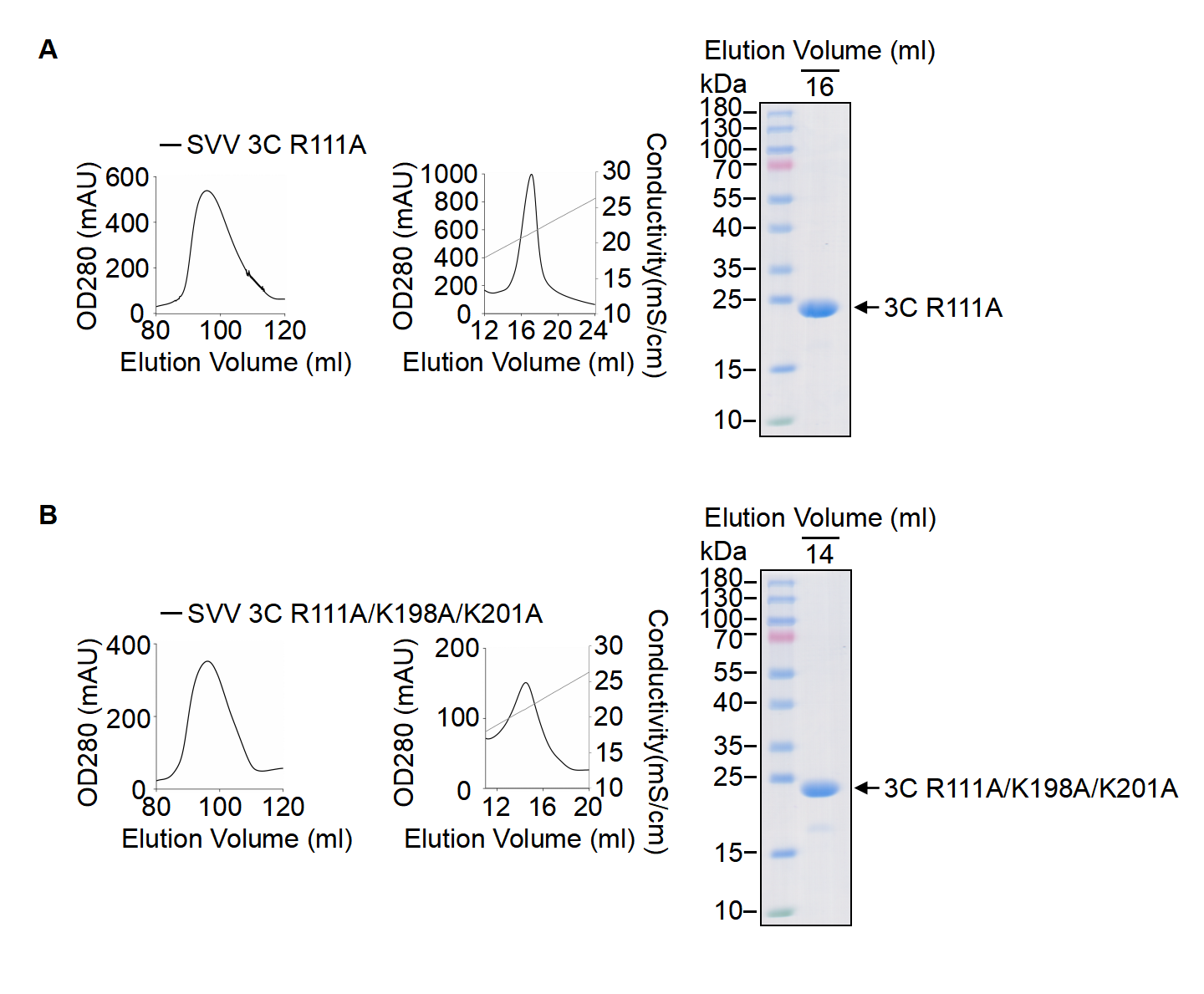


**A**-**B**, SUMO-SVV 3C R111A (**A**) and SUMO-SVV 3C R111A/K198A/K201A (**B**) recombinant proteins were cleaved by SUMO protease overnight at 4 °C. Subsequently, these mutant recombinant proteins were purified by FPLC and ion exchange, followed by SDS-PAGE analysis.

**Figure S8. SVV protease 3C cleaves nuclear cGAS.**


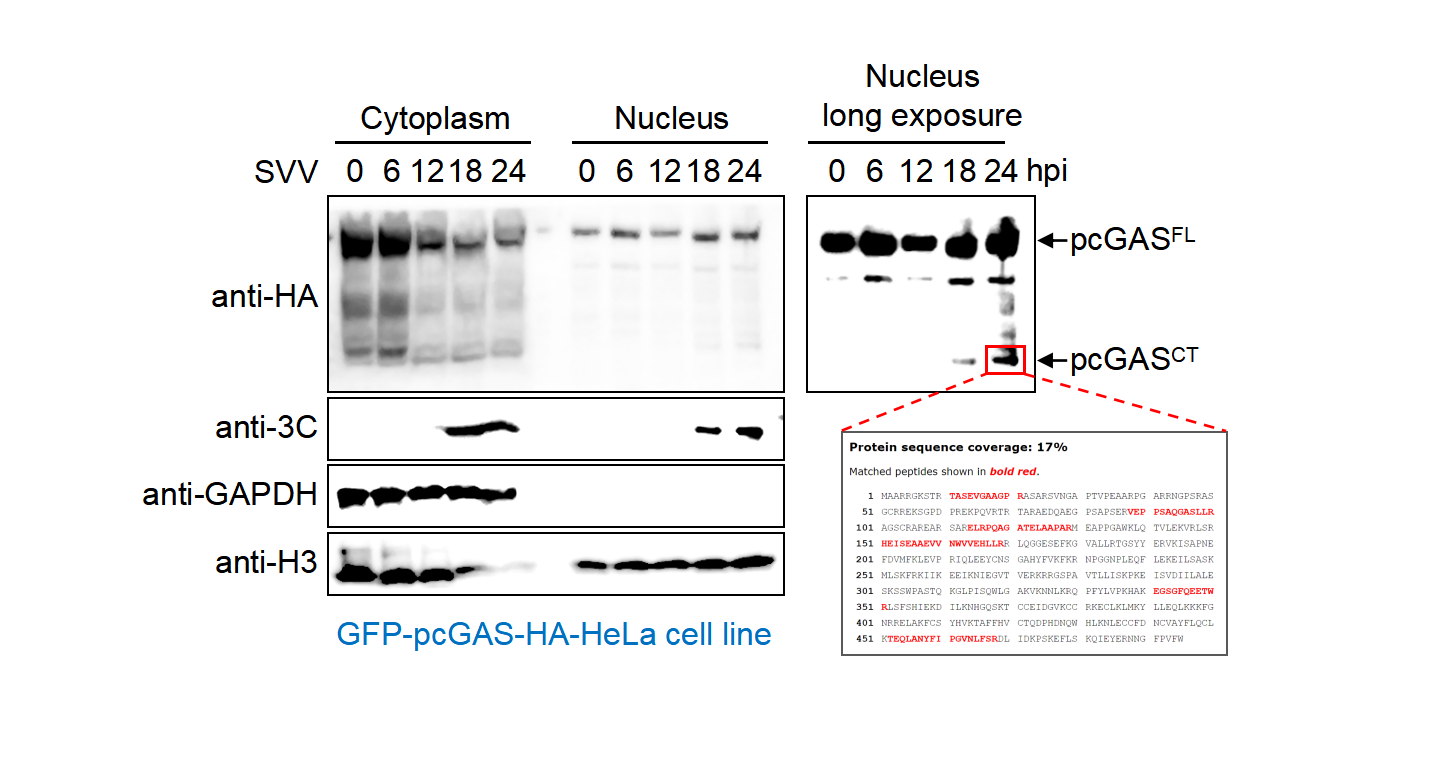


Detection of pcGAS cleavage in the nucleus of HeLa cells stably expressing GFP-pcGAS-HA, infected with SVV at MOI of 10 for 0, 6,12, 18 and 24 h, was performed by western blot analysis. Additionally, the pcGAS^CT^ band excised from the SDS-PAGE gel was subjected to protein sequence analysis via mass spectrometry.

**Figure S9. The localization and expression of pcGAS and pcGAS^CT^.**


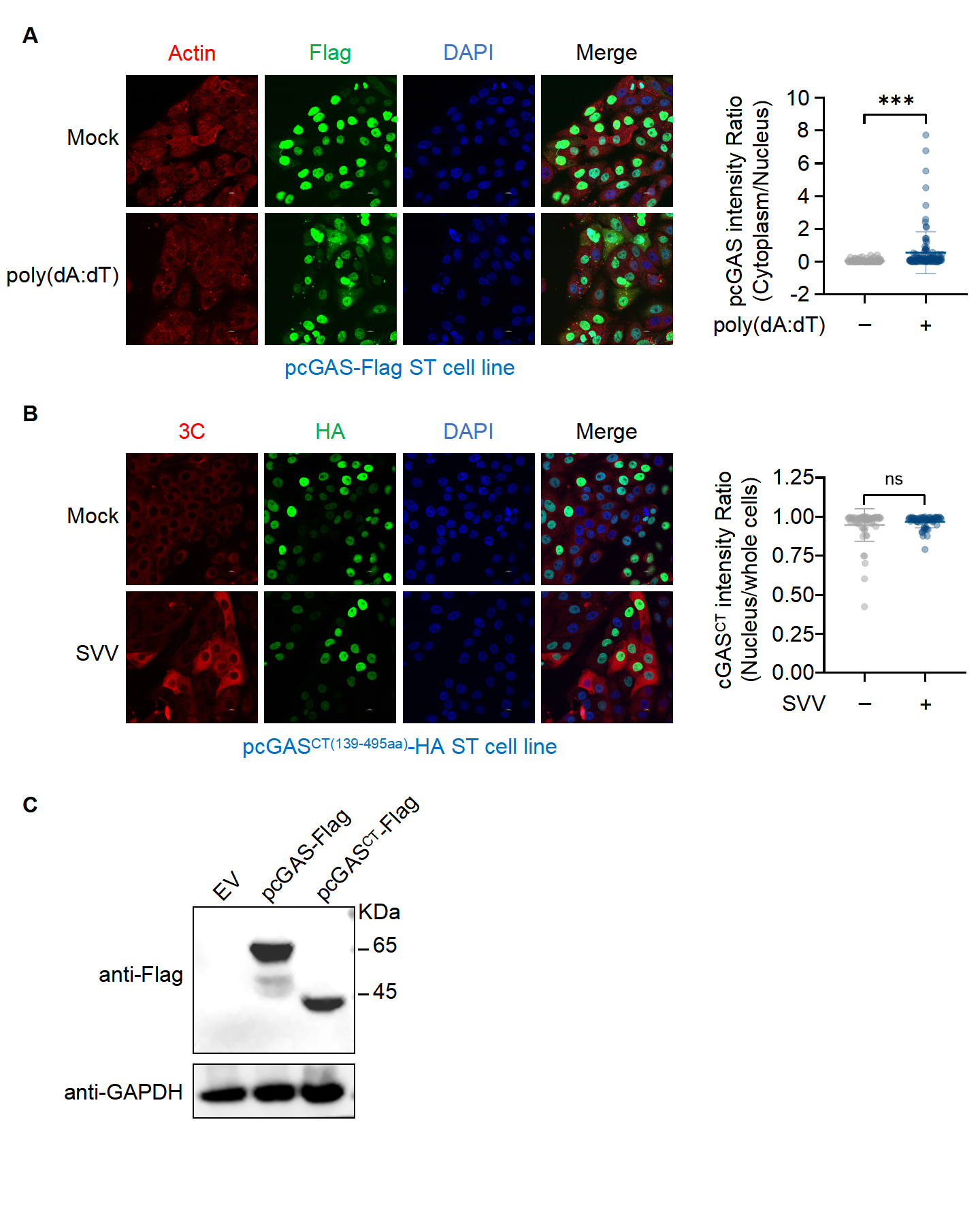


**A**, The re-localization of pcGAS in ST cells stably expressing pcGAS-Flag (pcGAS-Flag ST cell line) after mock-transfection or transfection with poly(dA:dT) for 6 h. Scale bars, 10 µm. **B**, The localization of pcGAS C-terminus in ST cells stably expressing pcGAS^CT(139-495aa)^-HA (pcGAS^CT^-HA-ST cell line) after mock-infection or infection with SVV (MOI 10) for 8 h. Scale bars, 10 µm. **C**, Western blot analysis of protein expression. HEK-293T cells were transfected with 1 µg of either pcGAS-Flag or pcGAS^CT^-Flag plasmids, and samples were collected 24 h later.

**Figure S10. The 3C (∆1-20aa) mutant loses its ability to cleave pcGAS.**


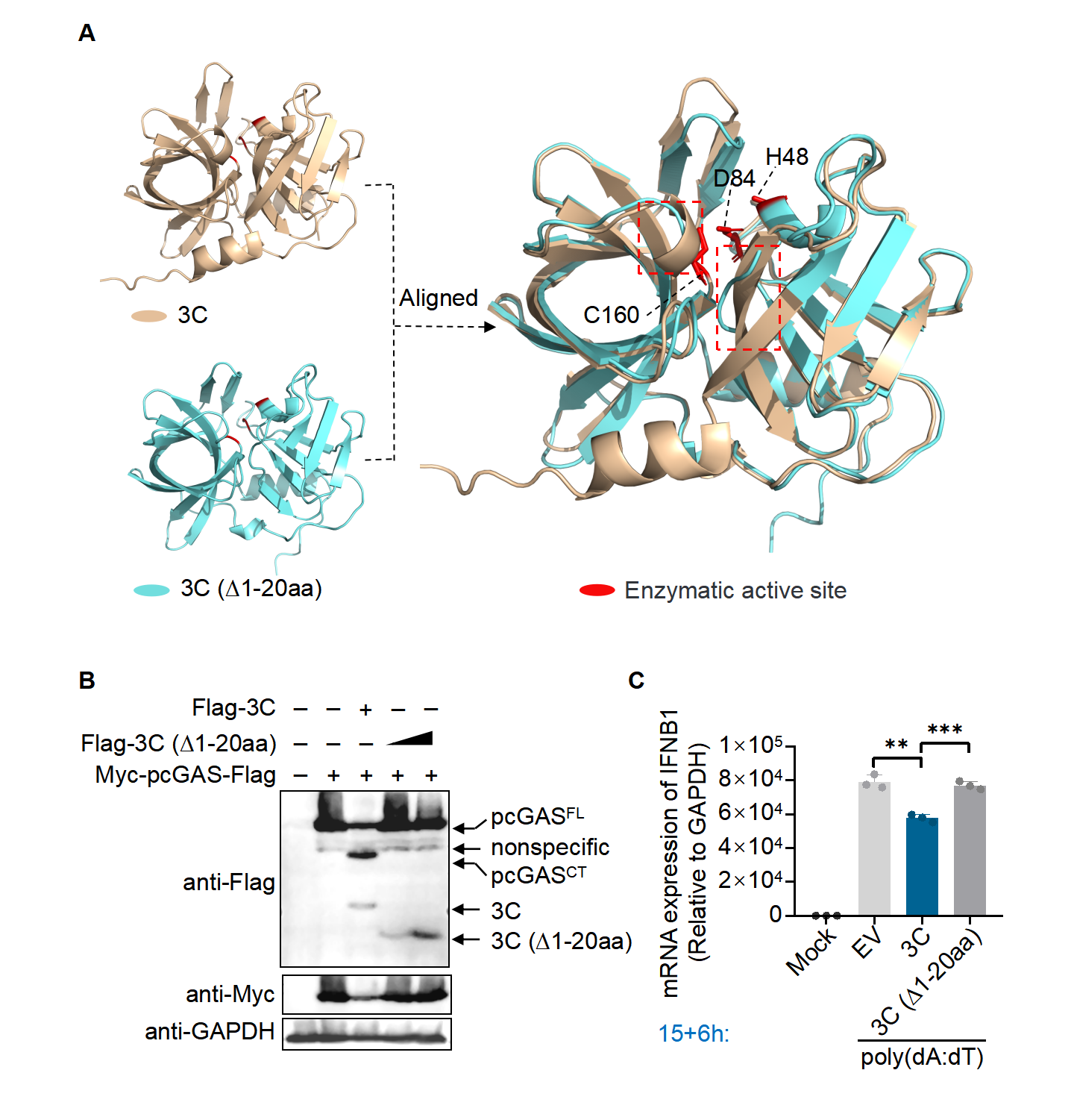


**A**, The protein structure of SVV 3C (∆1-20 aa) was predicted using AlphaFold3 and aligned with the wild-type 3C structure. The red box highlights the region where the conformational change occurred. **B**, HEK-293T cells were co-transfected with Myc-pcGAS-FLag plasmid along with Flag-3C or Flag-3C (∆1-20 aa) for 24 h followed by western blot analysis. **C**,ST cells were transfected with plasmids encoding either 3C and 3C (∆1-20 aa) for 15 h, followed by stimulation ith poly(dA:dT) for 6 h. Samples were then collected for qRT-PCR analysis to detect IFN-β expression.

**Table**

**Table S1. Simulated data on the binding of 3C protein to dsDNA.**


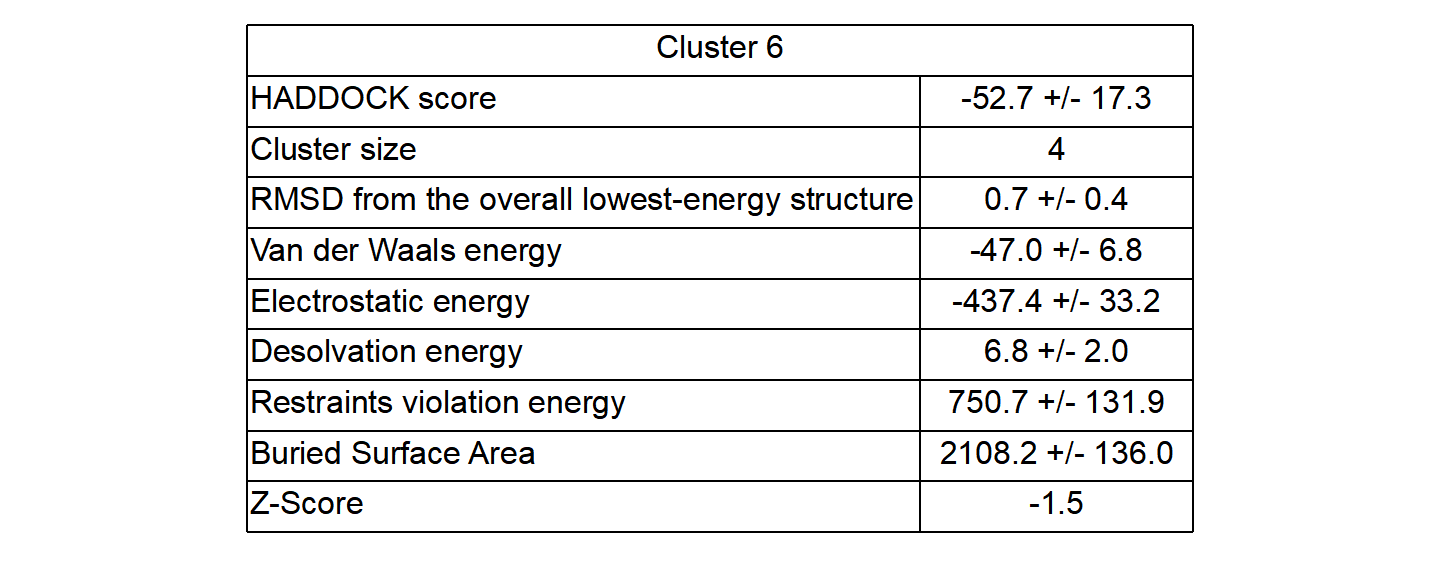


Simulation of SVV 3C binding to DNA was performed using HADDOCK2.4, with the parameters of the optimal model from cluster 6.
